# One-pot three-component tandem annulation of 4-hydroxycoumarine with aldehyde and aromatic amines using graphene oxide as an efficient catalyst

**DOI:** 10.1038/s41598-021-99360-3

**Published:** 2021-10-06

**Authors:** Rabindranath Singha, Aminul Islam, Pranab Ghosh

**Affiliations:** Department of Chemistry, University of North Bengal, Dist-Darjeeling, West Bengal India

**Keywords:** Chemistry, Catalysis, Organocatalysis

## Abstract

A convenient and efficient solvent-free, facile, one-pot three-component graphene oxide catalysed approach has been described for the synthesis of chromeno-[4,3-*b*]quinolin-6-one derivatives from 4-hydroxycoumarin with aldehydes and aromatic amines. Graphene oxide (GO) has proved to be a new class of heterogeneous carbocatalyst which could be easily recovered and reused up to 5th run without significant loss of its catalytic activity. A broad scope of substrate applicability is offered and a plausible mechanism is also suggested for this developed protocol.

## Introduction

Nowadays, rapid synthesis of highly important heterocyclic compounds becomes a great challenging task for synthetic chemists. Multi-component reaction (MCRs) is a great idea for success in this purpose. MCRs are the great reaction, in that three or more reactants join to generate the desired product^1–4^. MCRs has unique advantages such as low cost, high atom-economy, energy saving, smaller reaction time and cheap purification processes^5^. Chromeno-[4,3-*b*]quinolin-6-ones are one of the important heterocyclic compound because of the presence of this moiety in various natural product as well as in synthetic product. This heterocyclic compounds are also important in the application of medicine and optoelectronics^6^. Chromeno-quinoline derivatives are also widely used in medicinal chemistry as glucocorticoid^7^, selective progesterone receptor modulator^8^, estrogen receptor^9^, anti-inflammatory^10^, bacteriostatic activities^11^ and anti-cancer^12^. Though of their wide range of applicability’s, few of the synthetic procedure ever has been reported^13–16^. However, most of these procedures suffer from several disadvantages such as expensive catalyst, environmentally hazardous reagents and high reaction temperature. Keeping these views in mind we applied Graphene oxide (GO), as a greener carbocatalyst. Graphene oxide is a two dimensional thin layer nanostructure and it has unique chemical properties, high mechanical and thermal resistance^17,18^. It has several advantages such as low production cost, large-scale production and easy processing. It is also used as a precursor for the preparation of reduced graphene oxide (RGO)^19^. In recent years, scientists have found that GO has excellent properties with rich active oxygen-containing functional groups^20^. These oxygen-containing functional groups can be used as catalytic active centres for covalent/non-covalent modification design according to the requirements for specific application. On the other hand, the presence of such oxygen-containing functional groups also broadens the interlayer gap of graphene oxide. Considering the above we put GO as a catalyst in our reaction protocol. GO was prepared by modified Hummers method. GO contains different kind of oxygen containing functional groups like epoxide, carbonyl, hydroxyl, carboxyl^21^. Due to the presence of these different oxygenated functional groups in GO, it has been reported as moderate acidic and an oxidant catalyst in several reaction^22–26^. In the present study, we have developed a solvent free greener GO catalysed reaction for the synthesis of chromeno-[4,3-*b*]quinolin-6-ones using aniline, aldehyde and 4-hydroxycoumarine. This Graphene oxide (GO) catalysed reaction protocol has been reported for the first time. The reaction protocol was carried out under mild condition and has taken shorter reaction time and easily recovered the catalyst from the reaction mixture. The recovered catalyst was reused up to 5th cycle without loss of its catalytic activity. GO was characterised by HR-TEM, SEM, FT-IR analysis.

## Result and discussion

We started our explorative work by taking 4-hydroxycoumarine (1, 1 mmol), 4-methoxy benzaldehyde (2, 1 mmol) and 4-methyl aniline (3, 1 mmol) as starting materials. The model reaction was with 20 mg of GO at 130 °C under neat condition for the reaction and this was selected as optimized condition of the model reaction (Table 1, entry 2). When same reaction was carried out in absence of the GO catalyst at 130 °C under neat condition for 24 h and we did not get the expected product (Table 1, entry 1). The model reaction was repeated by taking various amount of graphene oxide as catalyst (Table 1) and we observed that 20 mg of GO exerted the best result (Table 1, entry 2). After that, the reaction was also carried out in ethanol and water solvent but no one beat the yield of the desired product as compared to the neat reaction condition (Table 1, entry 3, 4). Thus the greener solvent-free condition was chosen as the optimal medium of the reaction. The reaction was also carried out at room temperature under solvent free condition for 12 h, but we failed to get the corresponding product (Table 1, entry 13).The effect of temperature on the yield of the product was also investigated.. When the model reaction was carried out at 60 °C and 80 °C temperature we did not get the expected product (Table 1, entry 9, 10). However, on raising the reaction temperature the yield of the desired product gradually increases and we get the best result at 130 °C temperature (Table 1, entry 11, 12, 2). Beyond 130 °C the yield of the product did not increase significantly (Table 1, entry 6). So, 130 °C was chosen as optimized reaction temperature.

**Table 1:** Optimization of reaction parameters for the synthesis of Chromeno-[4,3-*b*]-quinolin-6-ones in the protocol^a^.


Entry	Catalyst (mg)	Solvent (10 mL)	Temperature (°C)	Time (h)	Yield (%)^d^ 4b
1	–^[b]^	-^[c]^	130	24	NR
**2**	**20**	**-**	**130**	**10**	**94**
3	20	H_2_O	reflux	10	40
4	20	EtOH	reflux	10	50
5	20	–	130	7	83
6	20	–	140	10	95
7	30	–	130	10	96
8	35	–	130	10	96
9	20	–	60	10	NR
10	20	–	80	10	NR
11	20	–	100	10	20
12	20	–	120	10	70
13	20	–	RT^e^	12	NR
14	15	–	130	10	60
15	10	–	130	10	40

^[a]^Reaction conditions: 4-hydroxycoumarine (1, 1 mmol), 4-methoxy benzaldehyde (2, 1 mmol), *p*-toludine (3, 1 mmol), GO (20 mg) at different temperatures,^[b]^ In absence of catalyst,^[c]^ Under solvent-free condition.^[d]^Isolated yields, ^[e]^room temperature reaction, NR = no yield of the desired product.

To examine the applicability and generality of the model reaction, a variety of anilines and aldehydes with different electron donating/electron withdrawing groups were employed with 4-hydroxycoumarine. Anilines with electron donating groups afforded the desired product with high yield which might be due to the increased electron density in the aniline nucleus facilitating the ortho attack (Table 2, entries 4a-4 k, 4 m, 4n, 4p, 4q). However, when aniline with strong electron withdrawing group is used, we could not able to isolate the desired product instead traces amount of some other product was formed (identified by TLC). On the other hand, aromatic aldehydes with a variety of substituent groups furnished the corresponding product in good yields without any significant deviation. With aliphatic aldehydes, we get the desired product but the yield was comparatively low (Table 2, entries 4p, 4q). Heterocyclic aldehydes were also tried to determine the substrate scope of this reaction and the developed protocol afforded the desired product in good yield (Table 2, entries 4 k, 4o). From the results as listed in Table 2, it is obvious that different type of aldehydes and anilines can be successfully used and thus the developed protocol showed a wide range of substrate applicability.

**Table 2:**
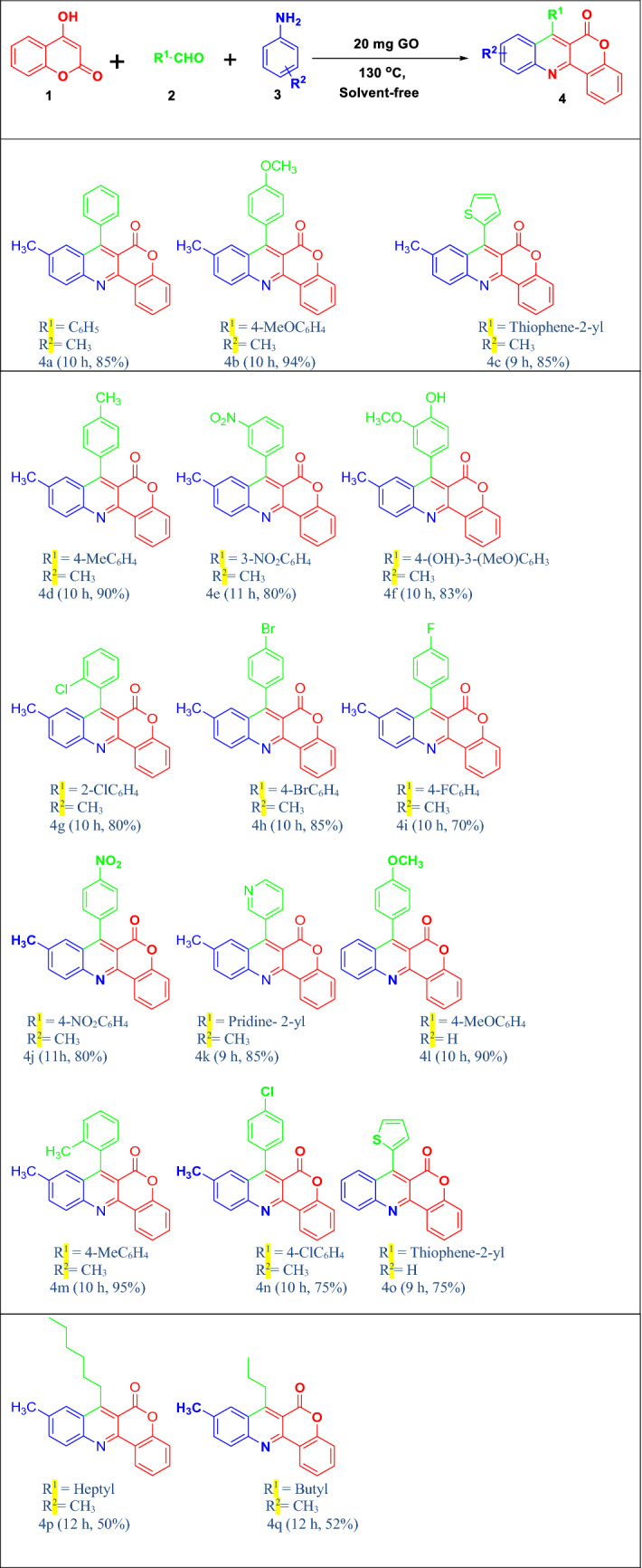
Substrate scope of anilines and aldehydes for the synthesis of chromeno-[4,3-*b*]-quinolin-6-ones in the protocol^a^.

We examined the catalytic activity of graphene oxide (GO) in the three component condensation reaction of aldehydes, aromatic amines and 4-hydroxycoumarine as an acid promoted catalyst. GO was synthesised by modified Hummer’s method and its surface consists of different oxygen containing functionalities namely epoxide, hydroxyl, carbonyl, carboxyl moieties. In FT-IR spectroscopy, the stretching frequencies of synthesised GO at 3400–3600 cm^−1^, 1720–1755 cm^−1^, 1605–1620 cm^−1^, 1230 cm^−1^ etc. which indicated the existence of these oxygenated functionalities in the synthesised GO. These results encourage us to use GO as solid heterogeneous acid catalyst for this reaction. HR-TEM and SEM images of GO showed the formation of layered structure of graphene oxide (GO) during its preparation.

**Figure 1:**
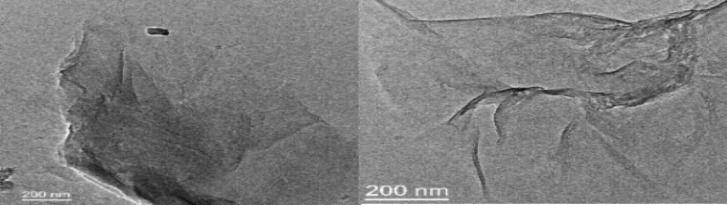
HR-TEM images of GO and GO after 5th run.

**Figure 2:**
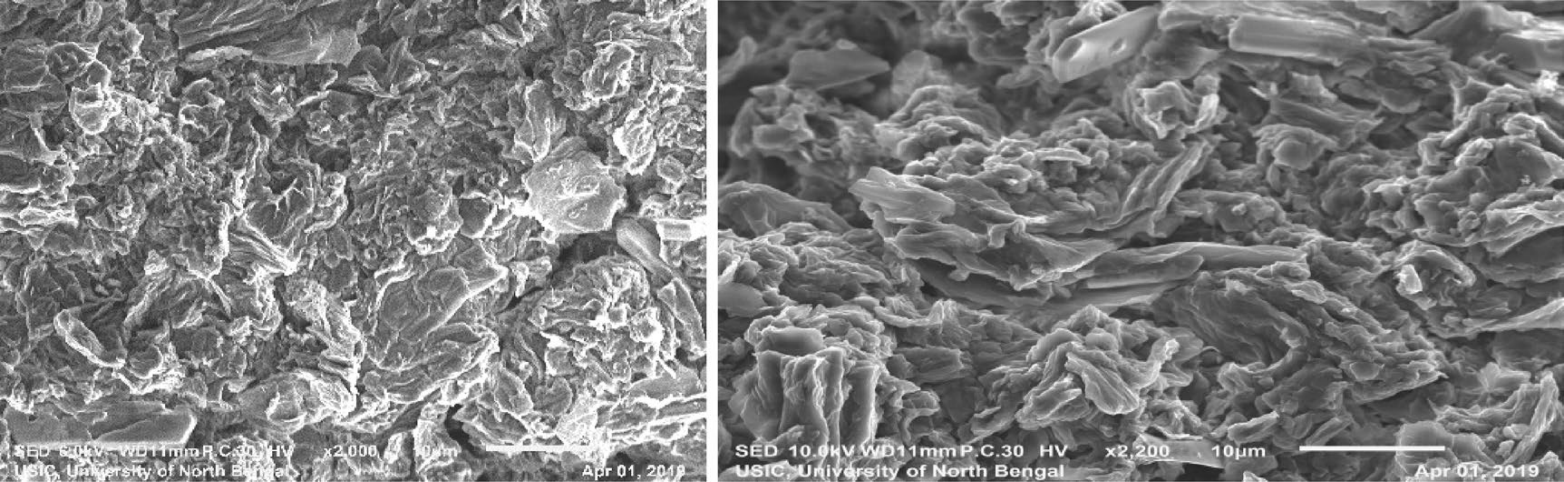
SEM images of GO and GO after 5th run.

Acidic functionalities of GO have taken part during the reaction which was strongly supported by the agglomeration and disintegration of GO into small layers after successive runs and this is distinctly predictable from the Figs. 1 and 2.

**Figure 3:**
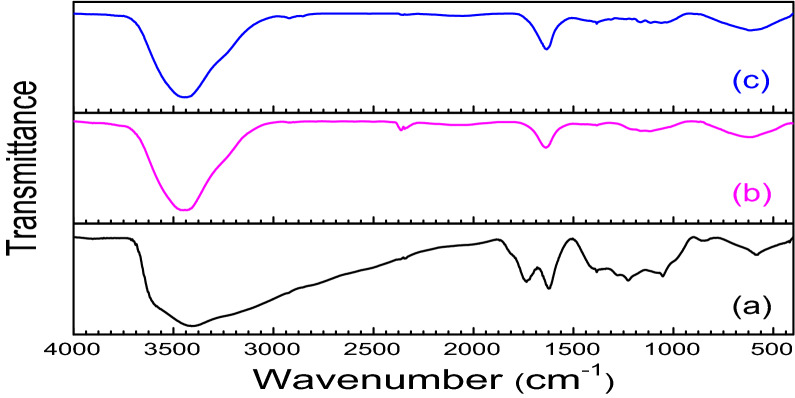
FT-IR spectra of (**a**) GO (**b**) after 2nd run and (**c**) after 5th run.

**Figure 4:**
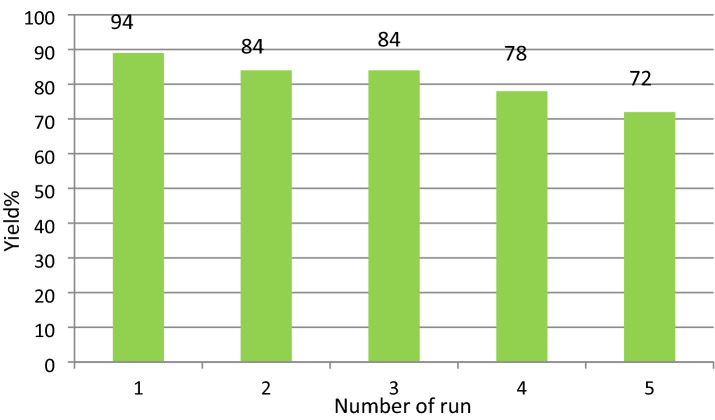
Recyclability of graphene oxide (GO).

FT-IR spectra of fresh GO and recovered GO after 2nd and 5th run was compared in order to explain the participation of acidic groups during the catalysis. The reduction of the band intensity of –OH and –COOH appeared at 3400–3600 cm^−1^ and 1720–1755 cm^−1^ respectively which significantly indicates the participation of these functional groups in reaction, the intensities of the bands gradually decreases (Fig. 3).The catalytic activity of GO is reduced after 5th run (Fig. 4) and this was happened may be due to the participation of oxygen containing acidic groups during the reaction.

**Scheme 1:**
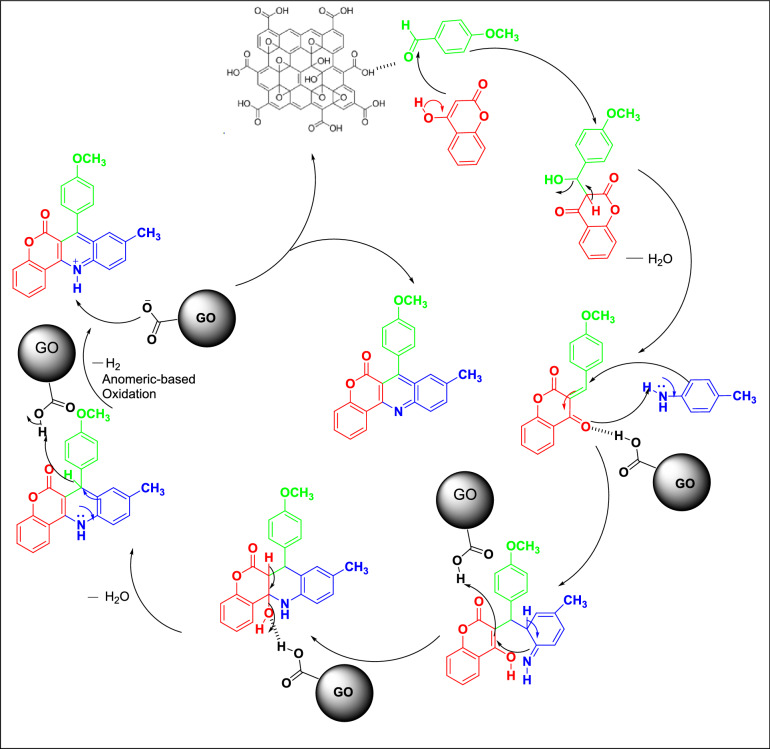
The plausible mechanism for the synthesis of chromeno-[4,3-b] quinolin-6-one derivatives.

We were highly encouraged to draw the plausible mechanism of the reaction (Scheme 1) catalysed by GO from the supportive instrumental results as well as from the earlier reports^25,27^. We assumed that, the first step of the reaction is the simple condensation between aldehyde and 4-hydroxycoumarine followed by the nucleophilic attack of *p*- toluidine on the unstable adduct. The adduct then rearranged through the transition intermediate and subsequently undergoes oxidation in the presence of GO and finally furnished the desired product.

## Conclusions

Herein, we have developed a simple, efficient and greener one-pot three component reaction protocol for synthesis of a variety of chromeno[4,3-*b*]quinolin-6-onesfrom aromatic amines, aldehydes and 4-hydroxycoumarine by using graphene oxide as a catalyst. The heterogeneous solid acid catalysts GO was found to be highly efficient for furnishing the corresponding products good to excellent yield. GO was cheap and easy recoverable catalyst and its catalytic activity sustained up to fifth run.

## Experimental section

### General information

All reagents were purchased from Sigma-Aldrich, TCI, Alfa-Aesar and used directly without further purification. The solvents were purchased from the commercial suppliers and used after distillation. All the synthesized products were purified by column chromatography on 60–120 mesh silica gels (SRL, India). For TLC, Merck plates coated with silica gel 60, F_254_ were used. IR spectra were recorded on KBr disc in the range 4000–400 cm^−1^ on Shimadzu FT-IR 8300 Spectrometer. ^1^H NMR and ^13^C NMR were recorded on 400 MHz Bruker-Avance FT-NMR Spectrometer using TMS as internal standard.

### General procedure for the preparation of GO by modified Hummer’s method

Graphene oxide (GO) was produced by modified hummers method from graphite powder. In this method, 180 mL of sulphuric acid (H_2_SO_4_) and 20 mL of phosphoric acid (H_3_PO_4_) with a volume ratio 9:1 were mixed and then 1.5 g of graphite powder was added to the solution in stirring condition. The temperature of the solution was kept below 20 °C and 9 g of potassium permanganate (KMnO_4_) was then added slowly into it. This mixture was stirred for 12 h. and small amount of hydrogen peroxide (H_2_O_2_) was added drop wise to eliminate excess KMnO_4_. 30 ml of 30% hydrochloric acid (HCl) and 200 mL of deionised water was added and centrifuged at 5000 rpm for 20 min. Then, the supernatant was decanted away and the residual was dried at 90 °C to get dry GO.

### General procedure for the synthesis of Chromeno[4,3-*b*]quinolin-6-ones

A 25 mL round-bottom flask was charged with 4-hydroxycoumarine (1, 1 mmol), 4-methoxy benzaldehyde (2, 1 mmol) and 4-methyl aniline (3, 1 mmol) then added graphene oxide (20 mg). The reaction mixture was stirred under neat condition at 130 °C temperature until consumption of the reactants indicated by TLC. After completion of the reaction the reaction mixture was partitioned between ethyl acetate and water and the catalyst was separated by simple filtration. The combined organic layer was dried over anhydrous sodium sulphate and concentrated under water bath. After that the products were purified by column chromatography on 60–120 mesh silica gels.

### 9-methyl-7-phenyl-6***H***-chromeno[4,3-***b***]quinolin-6-one (4a)^25^

White solid, m.p: 272–273 °C; IR (KBr):ν_max_ = 3019, 2945, 1722, 1631, 1194,999, 830, 750 cm^−1^; ^1^H NMR (400 MHz, CDCl_3_): 8.12 (s, 1H), 8.00–8.02 (m, 1H), 7.68–7.73 (m, 2H), 7.59 (s, 4H), 7.26–7.29 (m, 4H), 2.42 (s, 3H); ^13^C NMR (100 MHz, CDCl_3_): 21.87, 113.42, 117.94, 121.95, 124.18, 126.75, 127.05, 127.21, 127.82, 128.00, 128.13, 128.37, 135.69, 135.76, 136.25, 137.30, 147.32, 154.72, 155.44, 157.51, 178.28.

### 7-(4-methoxyphenyl)-9-methyl-6***H***-chromeno[4,3-***b***]quinolin-6-one (4b)^27^

Yellow solid. m.p: 246–248 °C. FT-IR (KBr): ν_max_ = 3050, 2923, 1745, 1602,1553, 1454, 1243, 1179, 990, 827, 750 cm^−1^; ^1^H NMR (400 MHz, CDCl_3_): 8.80 (dd, ^1^*J* = 8.0 Hz, ^2^*J* = 1.6 Hz, 1H), 8.10 (d, *J* = 8.4 Hz, 1H), 7.66 (dd, ^1^*J* = 8.6 Hz, ^2^*J* = 1.8 Hz, 1H), 7.52–7.56 (m, 1H), 7.37–7.41 (m, 1H), 7.30 (d, *J* = 8.4 Hz, 2H), 7.21–7.24 (m, 2H), 7.09–7.12 (m, 2H), 3.95 (s, 3H), 2.41 (s, 3H); ^13^C NMR (100 MHz, CDCl_3_): 159.72, 159.50, 154.76, 152.59, 149.30, 149.04, 137.28, 135.24, 132.02, 129.49, 129.29, 129.20, 128.40, 126.86, 125.58, 124.56, 120.05, 116.93, 113.88, 113.41, 55.40, 21.98.

### 9-methyl-7-(thiophen-2-yl)-6***H***-chromeno[4,3-***b***]quinolin-6-one (4c) ^25^

Yellow solid, m.p: ˃250 °C; IR (KBr): ν_max_ = 3022, 2925, 1745, 1664, 1212, 1176, 756 cm^−1^; ^1^H NMR (400 MHz, CDCl_3_): 8.81 (dd, ^1^*J* = 8.0 Hz, ^2^*J* = 1.6 Hz, 1H), 8.14 (d, *J* = 8.8 Hz, 1H), 7.71 (dd, ^1^*J* = 8.6 Hz, ^2^*J* = 1.8 Hz, 1H), 7.63–7.64 (m, 1H), 7.53–7.58 (m, 1H), 7.48 (s, 1H), 7.38–7.42 (m, 1H), 7.26–7.33 (m, 2H), 7.07–7.08 (m, 1H), 2.47 (s, 3H); ^13^C NMR (100 MHz, CDCl_3_): 159.11, 152.55, 149.28, 149.03, 147.49, 137.87, 136.55, 135.60, 132.19, 129.29, 129.00, 127.59, 127.24, 127.04, 126.38, 125.59, 124.66, 119.87, 116.97, 114.67, 22.06.

### 9-methyl-7-***p***-tolyl-6***H***-chromeno[4,3-***b***]quinolin-6-one (4d) ^27^

Pale yellow solid, m.p. : 209–211 °C, FT-IR (KBr): ν_max_ = 3050, 2965, 1752, 1670, 1550, 1455, 1265, 1187, 1097, 800 cm^−1^; ^1^H NMR (400 MHz, CDCl_3_): 8.83 (dd, ^1^*J* = 8.0 Hz, ^2^*J* = 1.6 Hz, 1H), 8.14 (d, *J* = 8.4 Hz, 1H), 7.69 (dd, ^1^*J* = 8.8 Hz, ^2^*J* = 1.6 Hz, 1H), 7.53–7.57 (m, 1H), 7.37–7.42 (m, 3H), 7.26–7.33 (m, 1H), 7.17 (d, *J* = 8 Hz, 2H), 2.52 (s, 3H), 2.43 (s, 3H); ^13^C NMR (100 MHz, CDCl_3_): 159.66, 155.08, 152.64, 149.35, 149.10, 137.81, 137.28, 135.30, 134.16, 132.05, 129.30, 129.30, 129.12, 128.24, 127.98, 126.87, 125.59, 124.57, 120.08, 116.97, 113.34, 21.97, 21.65.

### 9-methyl-7-(3-nitrophenyl)-6***H***-chromeno[4,3-***b***]quinolin-6-one (4e) ^27^

Yellow solid, m.p: > 250 °C; FT-IR (KBr): ν_max_ = 3093, 2920, 1743, 1598, 1554, 1530, 1461, 1349, 1180, 1104, 998, 829, 753 cm^−1^; ^1^H NMR (400 MHz, CDCl_3_): 8.86 (dd, ^1^*J* = 7.8 Hz, ^2^*J* = 1.4 Hz, 1H ), 8.17 (d, *J* = 8.4 Hz, 1H), 7.72 (dd, ^1^*J* = 8.8 Hz, ^2^*J* = 1.6 Hz, 1H), 7.54–7.59 ( m, 1H ), 7.32–7.48 (m, 5H), 7.15–7.26 (m, 1H), 7.06 (d, *J* = 7.2 Hz, 1H), 2.43 (s, 3H); ^13^C NMR (100 MHz, CDCl_3_): 159.43, 154.65, 152.67, 149.48, 149.28, 137.61, 136.91, 135.52, 135.02, 132.13, 130.04, 129.46, 128.31, 127.70, 127.53, 126.27, 125.91, 125.55, 124.64, 120.08, 117.04, 113.55, 22.00.

### 7-(4-hydroxy-3-methoxyphenyl)-9-methyl-6*H*-chromeno[4,3-*b*]quinolin-6-one (4f)

Yellow solid, m.p: ˃250 °C. FT-IR (KBr): ν_max_ = 3053, 2925, 1746, 1602, 1553, 1454, 1244, 1179, 990, 827, 755 cm^−1^; ^1^H NMR (400 MHz, CDCl_3_): 8.83 (dd, ^1^*J* = 7.8 Hz, ^2^*J* = 1.4 Hz, 1H), 8.14 (d, *J* = 8.8 Hz, 1H), 7.70 (dd, ^1^*J* = 8.6 Hz, ^2^*J* = 1.8 Hz, 1H), 7.53–7.58 (m, 1H), 7.37–7.43 (m, 2H), 7.26–7.33 (m, 1H), 7.12 (d, *J* = 8.0 Hz, 1 Hz), 6.77–6.80 (m, 2H), 5.83 (s, 1H), 3.88 (s, 3H), 2.45 (s, 3H); ^13^C NMR (100 MHz, CDCl_3_): 159.57, 154.75, 152.61, 149.36, 149.14, 146.68, 145.62, 137.37, 135.33, 132.08, 129.30, 128.90, 128.45, 126.92, 125.61, 124.60, 121.33, 120.05, 116.94, 114.50, 113.41, 111.11, 56.13, 22.01; MS (HRMS) (M + H)^+^ Calcd. for C_24_H_17_NO_4_: 383.1158; found: 384.1231.

### 7-(2-chlorophenyl)-9-methyl-6*H*-chromeno[4,3-*b*]quinolin-6-one (4 g)

Yellow solid, m.p: 243–245 °C; IR (KBr): 3020, 1738, 1609, 1216 cm-1; ^1^H NMR (400 MHz, CDCl_3_): 8.84 -8.86 (m, 1H), 8.17 (d, *J* = 8.8 Hz, 1H), 7.71–7.73 (m, 1H), 7.40–7.63 (m, 5H), 7.16–7.37 (m, 3H), 2.44 (s, 3H); ^13^C NMR (100 MHz, CDCl_3_): 159.44, 152.60, 151.31, 149.45, 149.35, 137.88, 136.31, 135.63, 132.37, 132.19, 131.64, 130.46, 129.71, 129.67, 129.55, 129.41, 127.30, 126.95, 125.88, 125.54, 124.71, 119.98, 117.07, 113.67, 22.05; MS (HRMS) (M + H)^+^ Calcd for C_23_H_14_ClNO_2_: 371.0713; found: 372.0788.

### 7-(4-bromophenyl)-9-methyl-6***H***-chromeno[4,3-***b***]quinolin-6-one (4 h) ^25^

White solid, m.p: 259–251 °C; IR (KBr): ν_max_ = 3025, 2917, 1720, 1604, 1223, 1167, 1073, 767 cm^−1^; ^1^H NMR (400 MHz, CDCl_3_): 8.82 (dd, ^1^*J* = 7.8 Hz, ^2^*J* = 1 Hz, 1H), 8.15 (d, *J* = 8.4 Hz, 1H), 7.70 (dd, ^1^*J* = 8.6 Hz, ^2^*J* = 1.8 Hz, 3H), 7.54–7.58 (m, 1H), 7.40–7.43 (m, 1H), 7.32 (d, *J* = 8.0 Hz, 1H), 7.16–7.21 (m, 3H), 2.44 (s, 3H); ^13^C NMR (100 MHz, CDCl_3_): 159.67, 153.23, 152.57, 149.31, 149.17, 137.74, 136.16, 135.53, 132.22, 131.67, 129.79, 129.45, 127.73, 126.39, 125.59, 124.72, 122.42, 119.90, 117.02, 113.15, 21.99.

### 7-(4-fluorophenyl)-9-methyl-6***H***-chromeno[4,3-***b***]quinolin-6-one(4i) ^25^

White solid, m.p: 234–236 °C; IR (KBr): 3030, 2935, 1719, 1646, 1183, 985, 762 cm^−1^; ^1^H NMR (400 MHz, CDCl_3_): 8.83 (dd, ^1^*J* = 7.6 Hz, ^2^*J* = 1.6 Hz, 1H), 8.15 (d, *J* = 8.8 Hz, 1H), 7.71 (dd, ^1^*J* = 8.8 Hz, ^2^*J* = 1 Hz, 1H), 7.56–7.57 (m, 1H), 7.41–7.43 (m, 1H), 7.31–7.33 (m, 1H), 7.23–7.27 (m, 5H), 2.44 (s, 3H); ^13^C NMR (100 MHz, CDCl_3_): 162.72 (d, *J* = 245.50 Hz, C-F), 161.49, 159.72, 153.66, 152.58, 149.34, 149.16, 137.62, 135.47, 132.99, 132.95, 132.19, 129.90, 129.81, 129.42, 128.09, 126.50, 125.60, 124.70, 119.95, 117.00, 115.72, 115.51, 113.37, 22.00.

### 9-methyl-7-(pyridin-3-yl)-6*H*-chromeno[4,3-*b*]quinolin-6-one (4 k)

Solid, m.p: 137–139 °C. FT-IR (KBr): ν_max_ = 3052, 2925, 1728, 1662, 1595, 1553,1181, 947, 823, 754 cm^−1^; ^1^H NMR (400 MHz, CDCl_3_): 8.80–8.85 (m, 2H), 8.54–8.55 (m, 1H), 8.18 (d, *J* = 8.8 Hz, 1H), 7.73 (dd, ^1^*J* = 8.6 Hz, ^2^*J* = 1.4 Hz, 1H), 7.66–7.67 (m, 1H), 7.52–7.57 (m, 2H), 7.42–7.44 (m, 1H), 7.32 (d, *J* = 8.4 Hz, 1H), 7.19 (s, 1H), 2.43 (s, 3H); ^13^C NMR(100 MHz, CDCl_3_): 159.85, 152.53, 150.52, 149.38, 149.34, 149.25, 148.30, 138.06, 135.92, 135.72, 133.33, 132.34, 129.59, 127.82, 126.09, 125.61, 124.83, 123.21, 119.82, 117.05, 113.51, 22.00; MS (HRMS) (M + H)^+^ Calcd for C_22_H_14_N_2_O_2_: 338.1055; found: 339.1128.

### 7-(4-methoxyphenyl)-6***H***-chromeno[4,3-***b***]quinolin-6-one (4 l) ^25^

Brown solid, m.p: 228–230 °C; IR(KBr): ν_max_ = 3849, 3740, 3672, 3394, 3010, 1743, 1613, 1553, 1515, 1492, 1464, 1405, 1243, 1215, 1175, 1031, 759, 668 cm^−1^; ^1^H NMR (400 MHz, CDCl_3_): 8.85 (s,1H), 8.24(s, 1H), 7.86–7.87 (m, 1H), 7.56–7.61(m, 2H), 7.41–7.49 (m, 2H), 7.33(s, 1H), 7.22(s, 2H), 7.10 (s, 2H), 3.93 (s, 3H); ^13^C NMR (100 MHz, CDCl_3_): 159.63, 159.59, 155.80, 152.78, 150.41, 150.18, 132.80, 132.32, 129.60, 129.51, 129.00, 128.48, 128.41, 127.07, 125.78, 124.63, 119.98, 117.00, 113.90, 113.46, 55.40.

### 9-methyl-7-o-tolyl-6*H*-chromeno[4,3-*b*]quinolin-6-one (4 m)

Yellow solid, m.p.: 195–197; FT-IR (KBr): ν_max_ = 3050, 2964, 1755, 1670, 1550, 1456, 1265, 1180, 1098, 8789 cm^−1^; ^1^H NMR (400 MHz, CDCl_3_): 8.85 (dd, ^1^*J* = 7.8 Hz, ^2^*J* = 1.4 Hz, 1H), 8.16 (d, *J* = 8.8 Hz, 1H), 7.70–7.72 (m, 1H), 7.54–7.58 (m, 1H), 7.32–7.48 (m, 5H), 7.15–7.26 (m, 1H), 7.06 (d, *J* = 7.6 Hz, 1H), 2.43 (s, 3H), 1.96 (s, 3H); ^13^C NMR (100 MHz, CDCl_3_): 159.44, 154.65, 152.66, 149.48, 149.28, 137.62, 136.92, 135.53, 135.02, 132.13, 130.04, 129.45, 128.32,127.70, 127.53, 126.27, 125.92, 126.55, 124.64, 120.08, 117.04, 113.55, 22.00, 19.88; MS (HRMS) (M + H)^+^ Calcd for C_24_H_17_NO_2_: 351.1259; found: 352.1330.

### 7-(4-chlorophenyl)-9-methyl-6***H***-chromeno[4,3-***b***]quinolin-6-one (4n) ^27^

Pale yellow solid, m.p: 245–247 °C; FT-IR (KBr): ν_max_ = 3045, 2912, 1723, 1596, 1548, 1489, 1177, 992, 834, 757 cm^-1^; ^1^H NMR (400 MHz, CDCl_3_ ): 8.82 (dd, ^1^*J* = 7.8 Hz, ^2^*J* = 1.0 Hz, 1H), 8.15 (d, *J* = 8.8 Hz, 1H), 7.70 (dd, ^1^*J* = 8.8 Hz, ^2^*J* = 1.6 Hz, 3H), 7.54–7.59 (m, 1H), 7.40–7.43 (m, 1H), 7.31–7.33 (m, 1H), 7.16–7.26 (m, 3H), 2.43 (s, 3H); ^13^C NMR(100 MHz, CDCl_3_): 159.68, 153.23, 152.58, 149.32, 149.18, 137.74, 136.16, 135.53, 132.22, 131.68, 129.79, 129.45, 127.73, 126.39, 125.53, 124.73, 122.42, 119.90, 117.02, 113.15, 21.99.

### 7-(thiophen-2-yl)-6***H***-chromeno[4,3-***b***]quinolin-6-one (4o)^27^

Pale yellow solid, m.p: > 250 °C; FT-IR (KBr): ν_max_ = 3090, 2920, 1737, 1616, 1550, 1490, 1190, 1098, 974, 757 cm^−1^; ^1^H NMR (400 MHz, CDCl_3_): 8.85 (dd, ^1^*J* = 7.6 Hz, ^2^*J* = 1.6 Hz, 1H), 8.25 (d,*J* = 8.4 Hz, 1H), 7.87–7.90 (m, 1H), 7.77(d, *J* = 8.4 Hz, 1H), 7.62–7.64(m, 1H), 7.54–7.58 (m, 2H), 7.40–7.44 (m, 1H), 7.33–7.35 (m, 1H), 7.25–7.28 (m, 1H), 7.07–7.08 (m, 1H); ^13^C NMR (100 MHz, CDCl_3_): 158.95, 152.74, 150.34, 150.13, 148.58, 136.32, 133.07, 132.47, 129.58, 129.04, 127.96, 127.75, 127.55, 127.21, 127.15, 125.79, 124.71, 119.80, 117.02, 114.73.

### 7-hexyl-9-methyl-6***H***-chromeno[4,3-***b***]quinolin-6-one (4p)^27^

White solid, m.p: 95–97 °C; FT-IR (KBr): ν_max_ = 3030, 2950, 2920, 2850, 1719, 1597, 1553, 1463, 1365, 1248, 1171, 833, 756 cm^−1^; ^1^H NMR (400 MHz, CDCl_3_): 8.71 (dd, ^1^*J* = 8.0 Hz, ^2^*J* = 1.6 Hz, 1H), 8.03 (d, *J* = 8.4 Hz, 1H), 7.95 (s, 1 Hz), 7.64 (dd, ^1^*J* = 8.6 Hz, ^2^*J* = 1.8 Hz, 1H), 7.46 (dd, ^1^*J* = 7.0 Hz, ^2^*J* = 1.4 Hz, 1H), 7.25–7.33 (m, 1H), 3.74 (t, *J* = 6.8 Hz, 2H), 2.55 (s, 3H), 1.65–1.67 (m, 2H), 1.51–1.59 (m, 2H), 1.30–1.35 (m, 4H), 0.82–0.88 (m, 3H); ^13^C NMR (100 MHz, CDCl_3_): 160.59, 158.26, 152.36, 149.54, 148.71, 137.12, 135.00, 131.91, 130.18, 127.37, 125.64, 124.54, 124.06, 120.16, 116.68, 113.36, 31.68, 30.93, 30.13, 29.52, 22.73, 22.28, 14.20.

## Supplementary Information

Supplementary Information.
