# Supplementary material for: One-pot three-component tandem annulation of 4-hydroxycoumarine with aldehyde and aromatic amines using graphene oxide as an efficient catalyst
Source: Sci Rep. 2021 Oct 6;11:19891. doi: 10.1038/s41598-021-99360-3 (PMC8494800; doi:10.1038/s41598-021-99360-3)
Supplement: Supplementary file 1 — Supplementary Information. [file 41598_2021_99360_MOESM1_ESM.pdf]

Supplementary Material

One-pot three-component tandem annulation of 4-hydroxycoumarine with aldehyde and aromatic amines using Graphene oxide as an efficient catalyst

Rabindranath Singha, Aminul Islam and Pranab Ghosh*

Department of Chemistry, University of North Bengal, Dist-Darjeeling, West Bengal, India.

Tel.: +91 (0353) 2776381; fax: +91 (0353) 2699001

E-mail address: pizy12@yahoo.com (P. Ghosh)

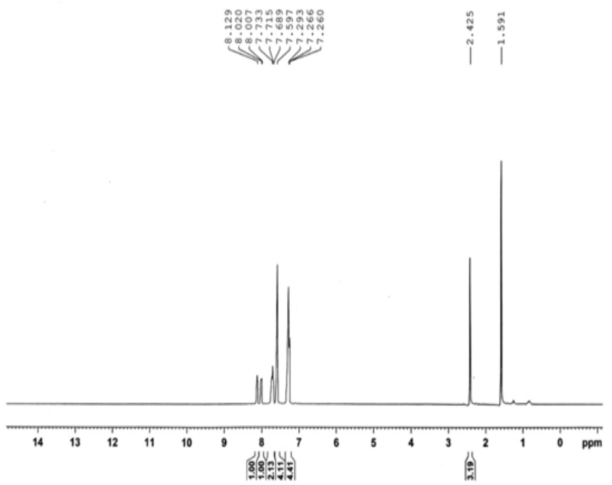

Fig 1. ^{1}H NMR spectrum of Compound 4a

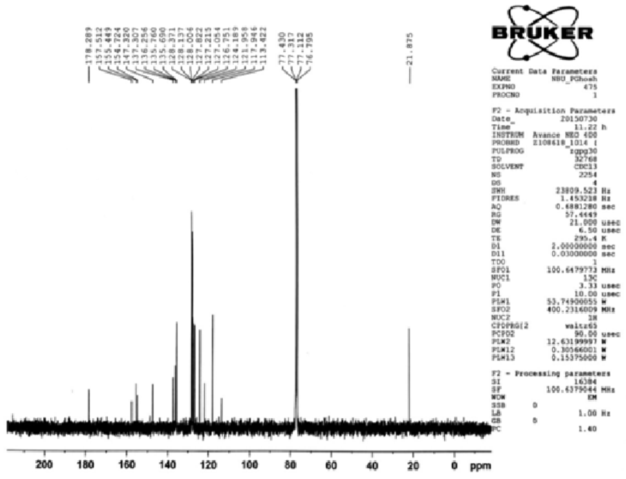

Fig 2. ^{13}C NMR spectrum of compound 4a

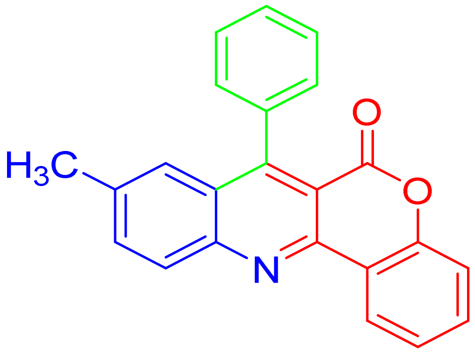

Fig 3. Structure of compound 4a

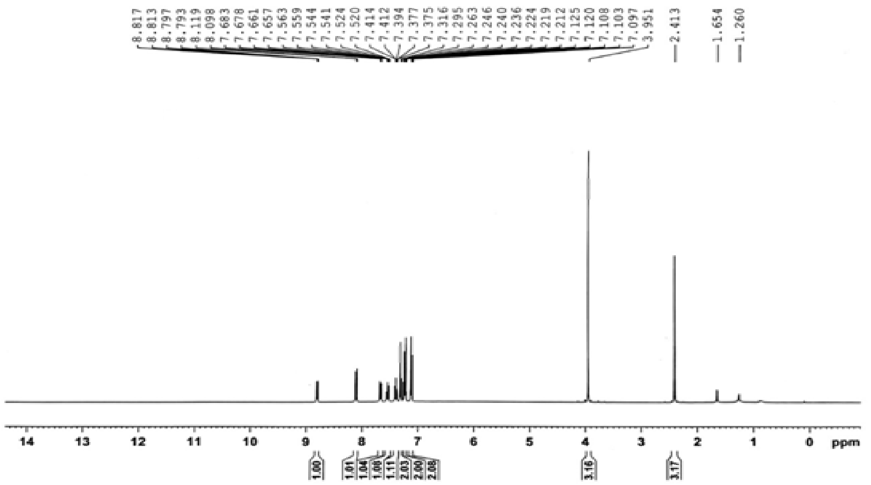

Fig 4. ^{1}H NMR spectrum of Compound 4b

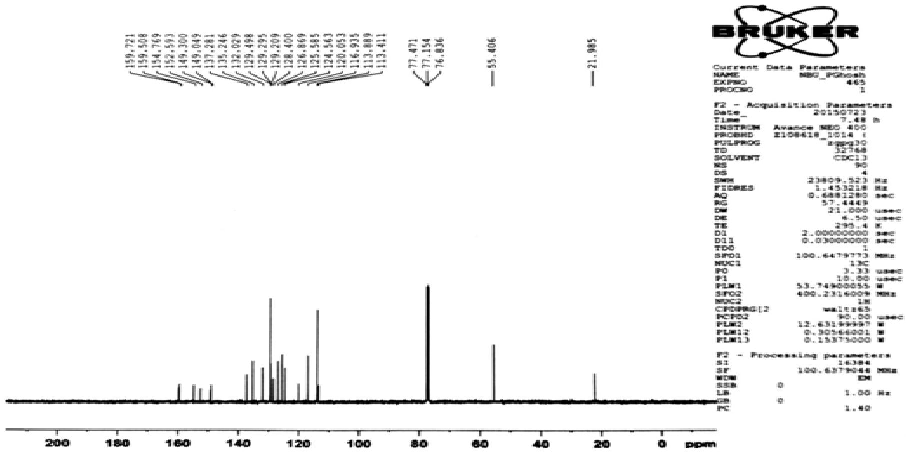

Fig 5. ^{13}C NMR spectrum of compound 4b

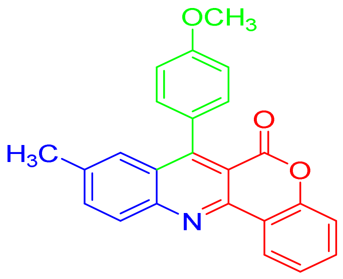

Fig 6. Structure of compound 4b

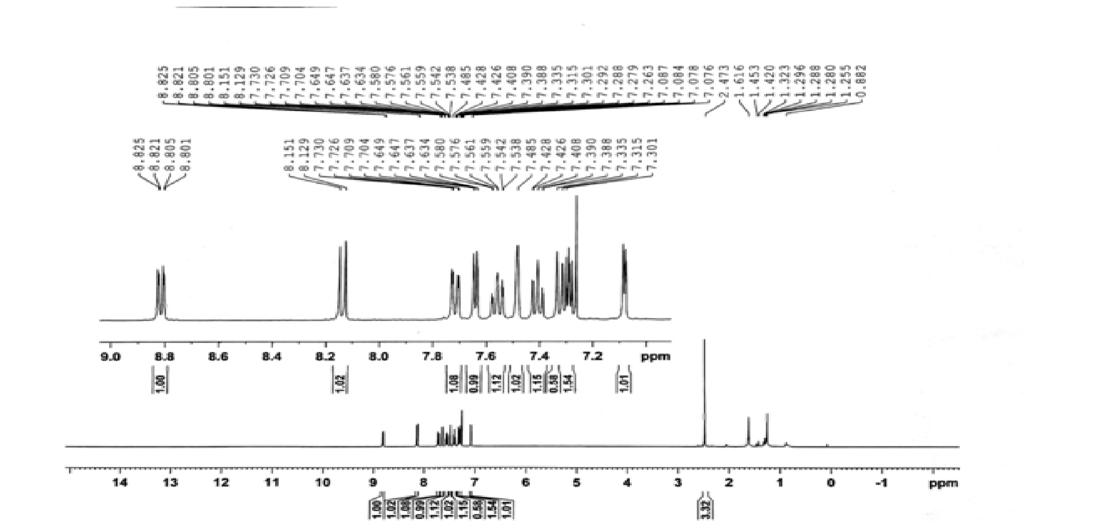

Fig 7. ^{1}H NMR spectrum of Compound 4c

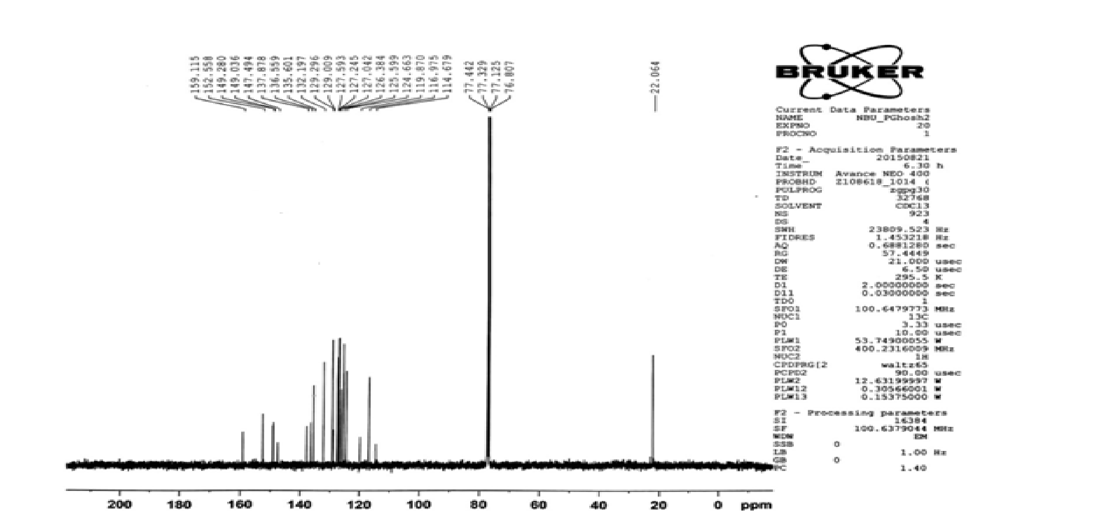

Fig 8. ^{13}C NMR spectrum of compound 4c

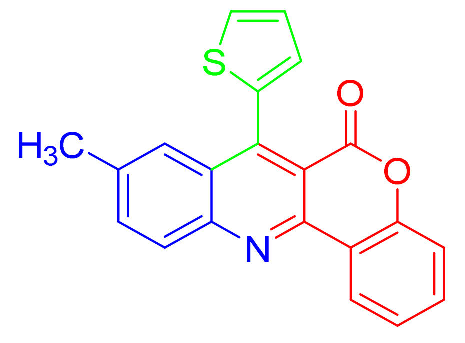

Fig 9. Structure of compound 4c

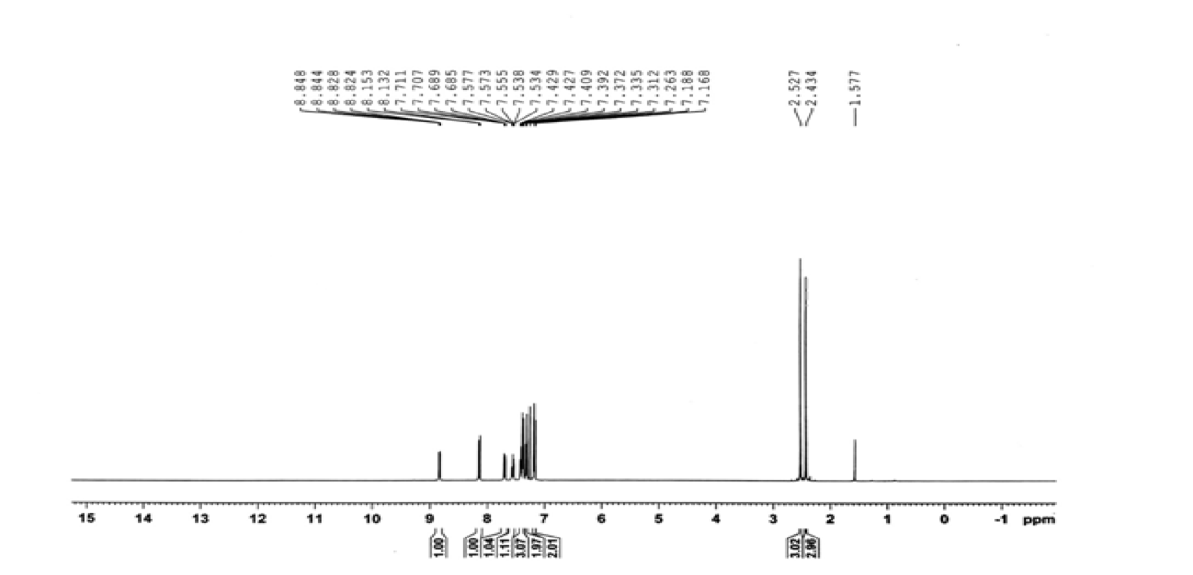

Fig 10. ^{1}H NMR spectrum of Compound 4d

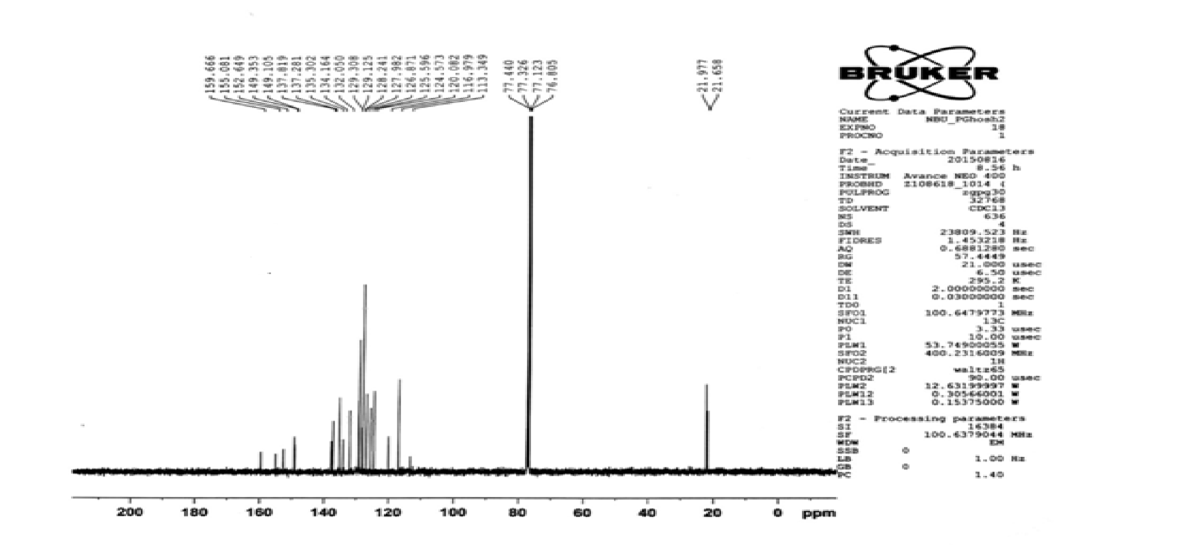

Fig 11. ^{13}C NMR spectrum of compound 4d

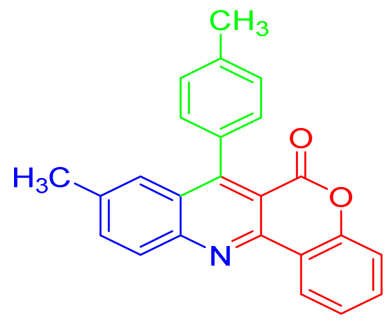

Fig 12. Structure of compound 4d

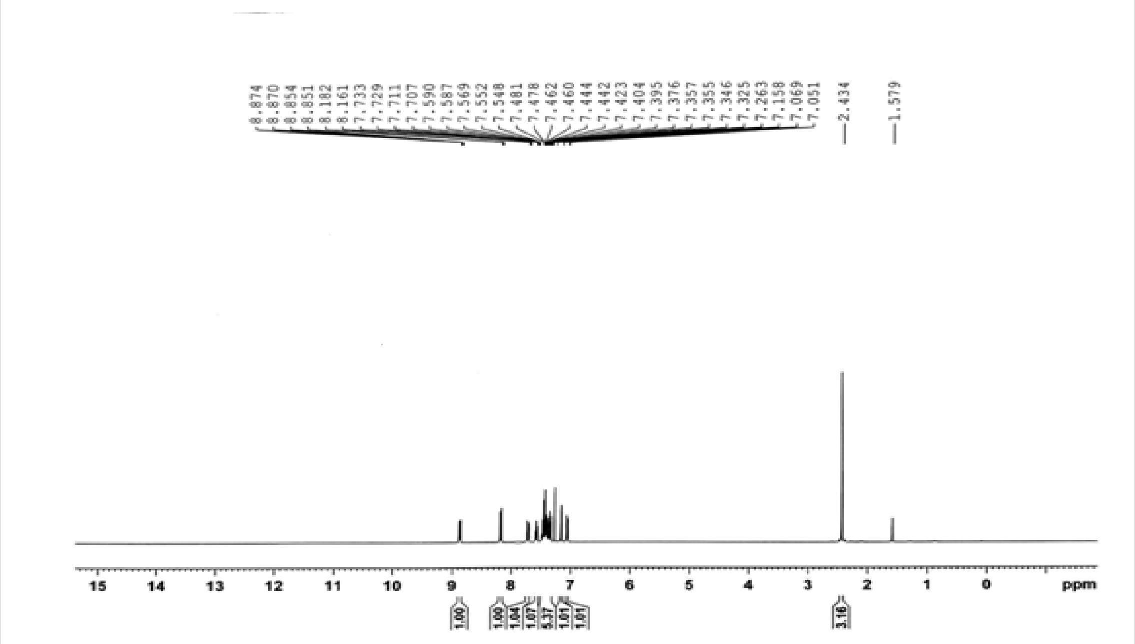

Fig 13. ^{1}H NMR spectrum of Compound 4e

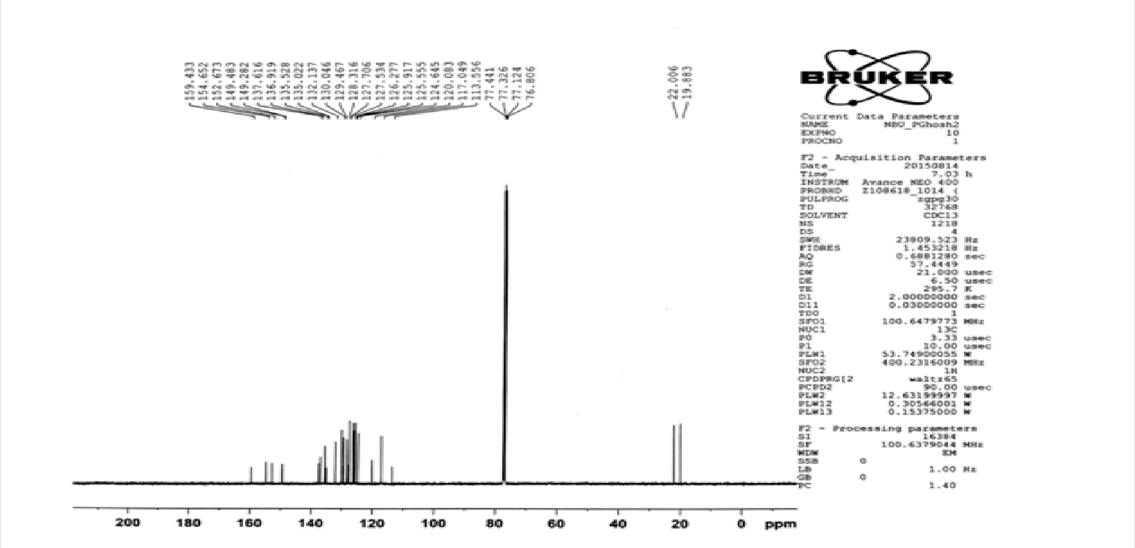

Fig 14. ^{13}C NMR spectrum of compound 4e

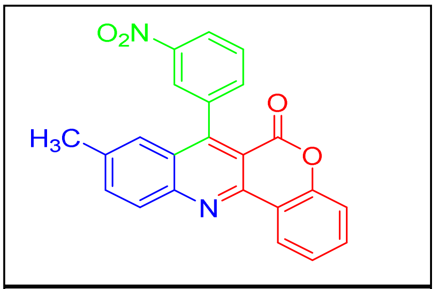

Fig 15. Structure of compound 4e

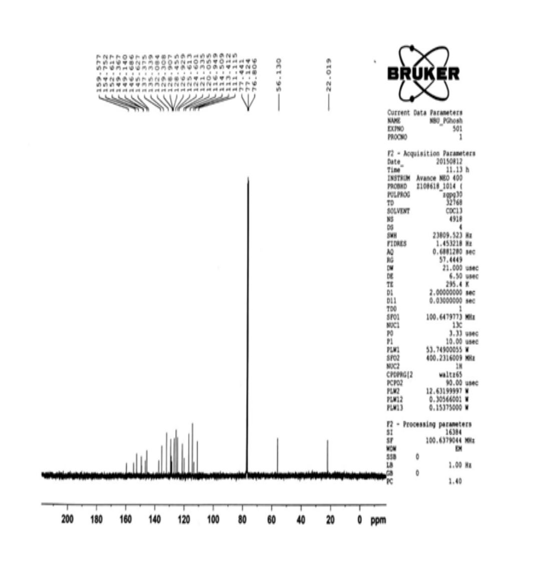

Fig 16. ^{13}C NMR spectrum of

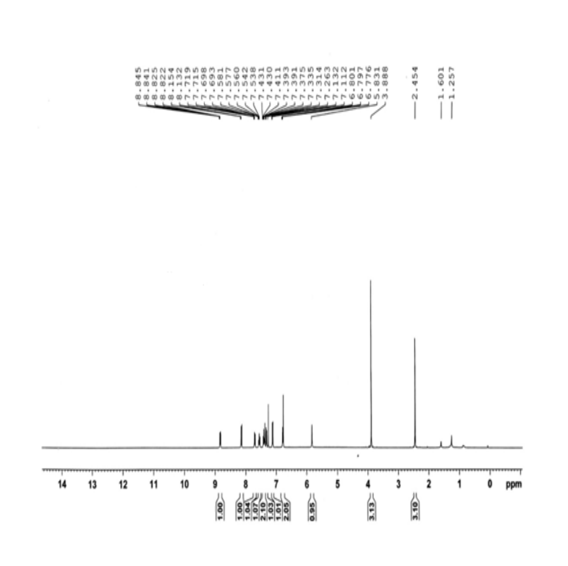

Fig 17. ^{1}H NMR spectrum of Compound

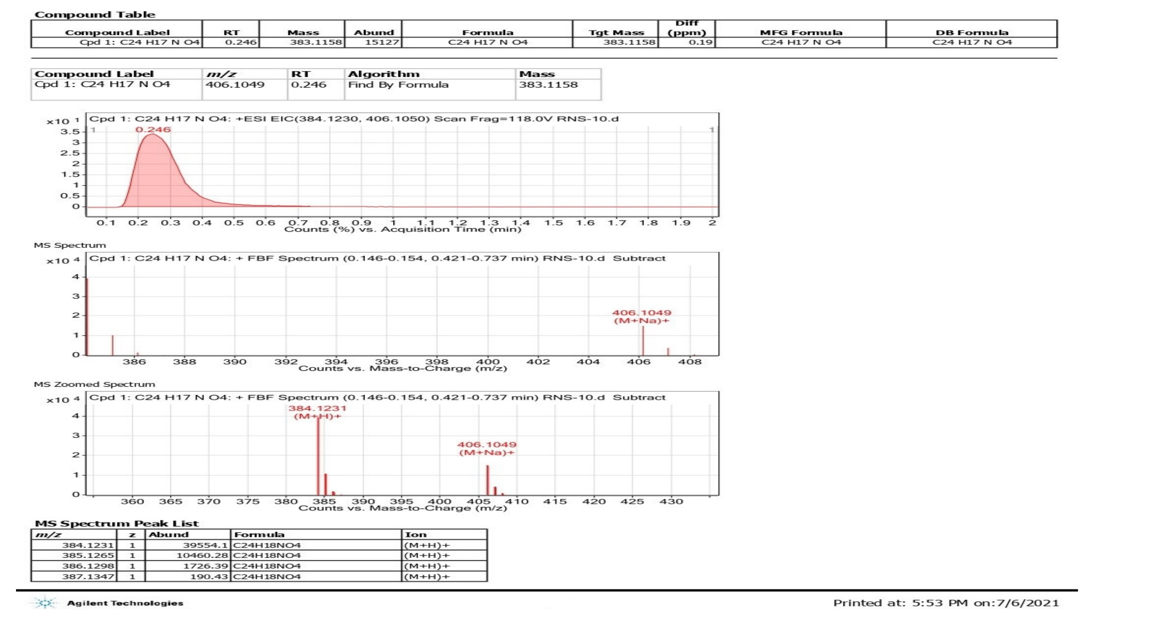

Compound Table

Compound Label	RT	Mass	Abund	Formula	Tgt Mass	Diff (ppm)	MFG Formula	DB Formula
Cpd 1: C24 H17 N O4	0.246	383.1158	15127	C24 H17 N O4	383.1158	0.19	C24 H17 N O4	C24 H17 N O4

Compound Label	m/z	RT	Algorithm	Mass
Cpd 1: C24 H17 N O4	406.1049	0.246	Find By Formula	383.1158

MS Spectrum Peak List

m/z	z	Abund	Formula	Ion
384.1231	1	39554.1	C24H18NO4	(M+H)+
385.1265	1	10460.28	C24H18NO4	(M+H)+
386.1298	1	1726.39	C24H18NO4	(M+H)+
387.1347	1	190.43	C24H18NO4	(M+H)+

Agilent Technologies

Printed at: 5:53 PM on:7/6/2021

Fig 18. Mass spectrum of Compound 4f

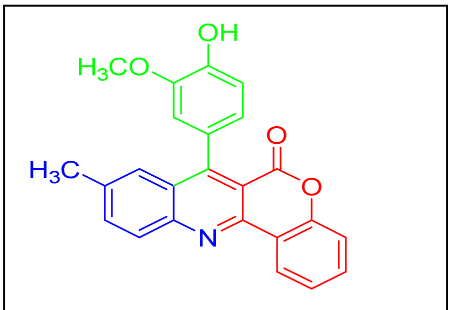

Fig 19. Structure of compound 4f

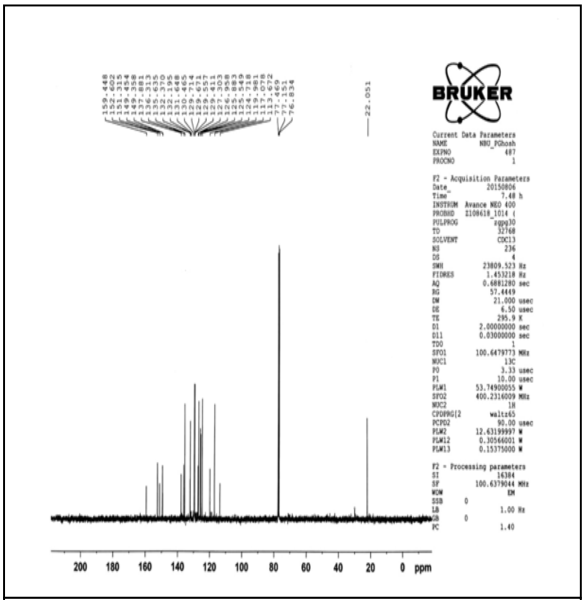

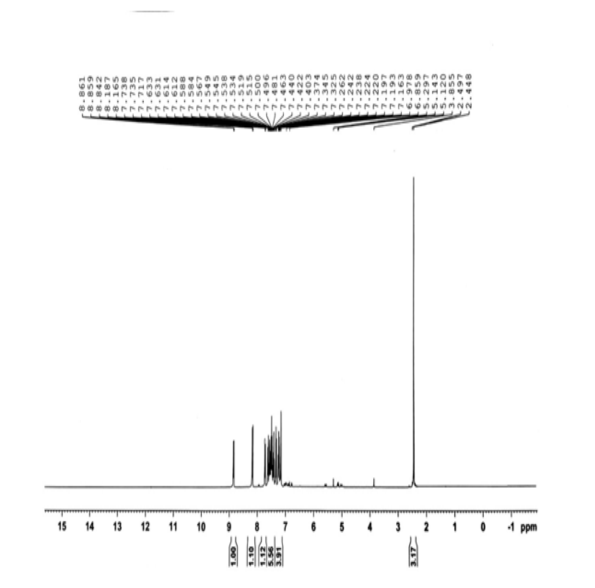

Fig 20. ^{13}C NMR spectrum of

Fig 21. ^{1}H NMR spectrum of

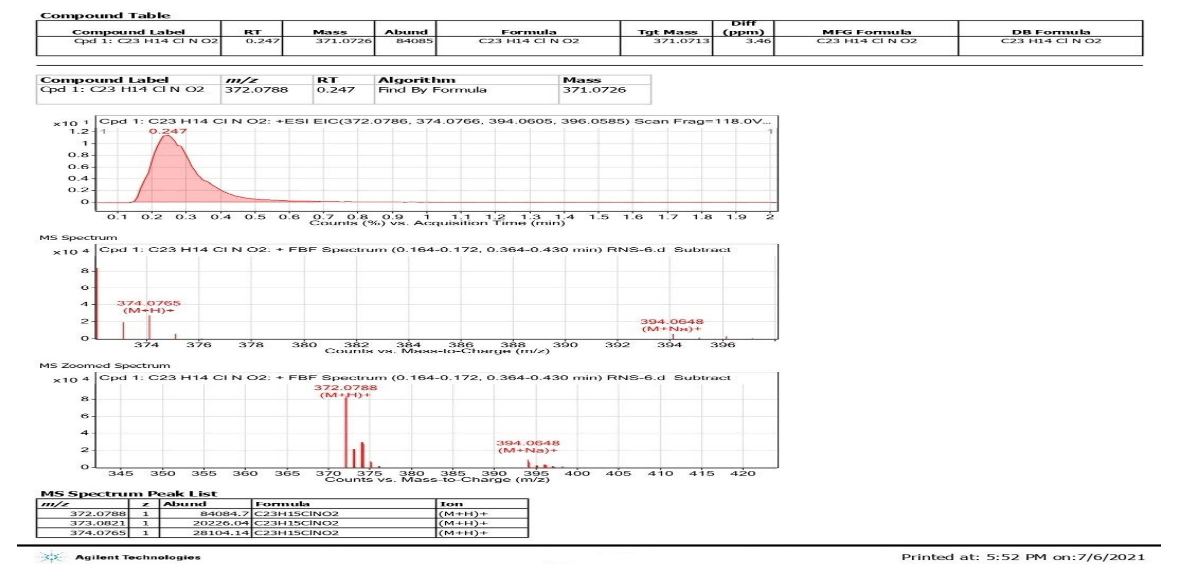

Fig 22. Mass spectrum of Compound 4g

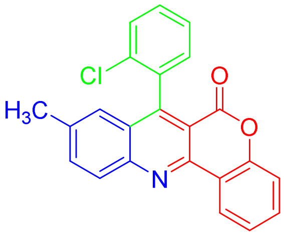

Fig 23. Structure of compound 4g

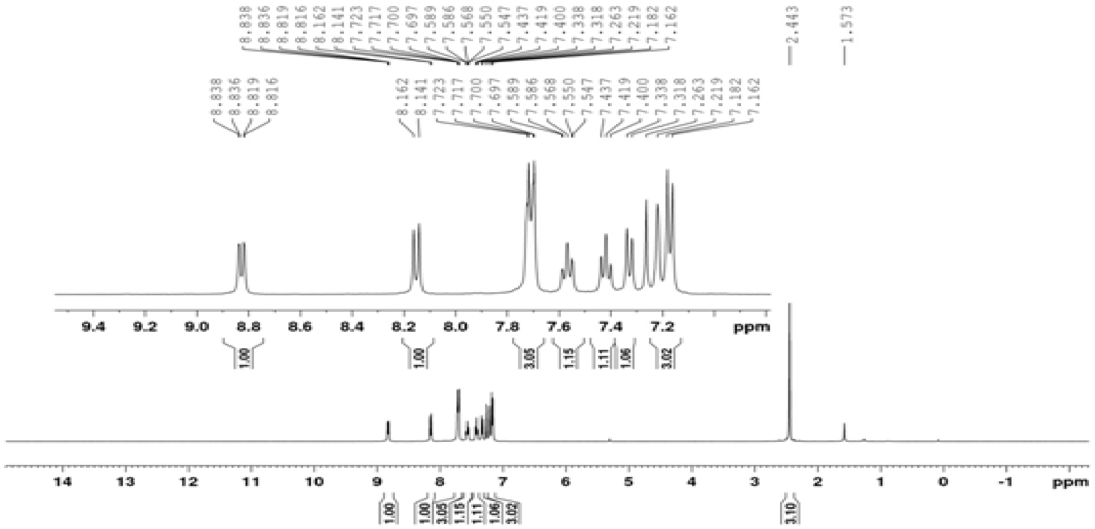

Fig 24. ^{1}H NMR spectrum of Compound 4h

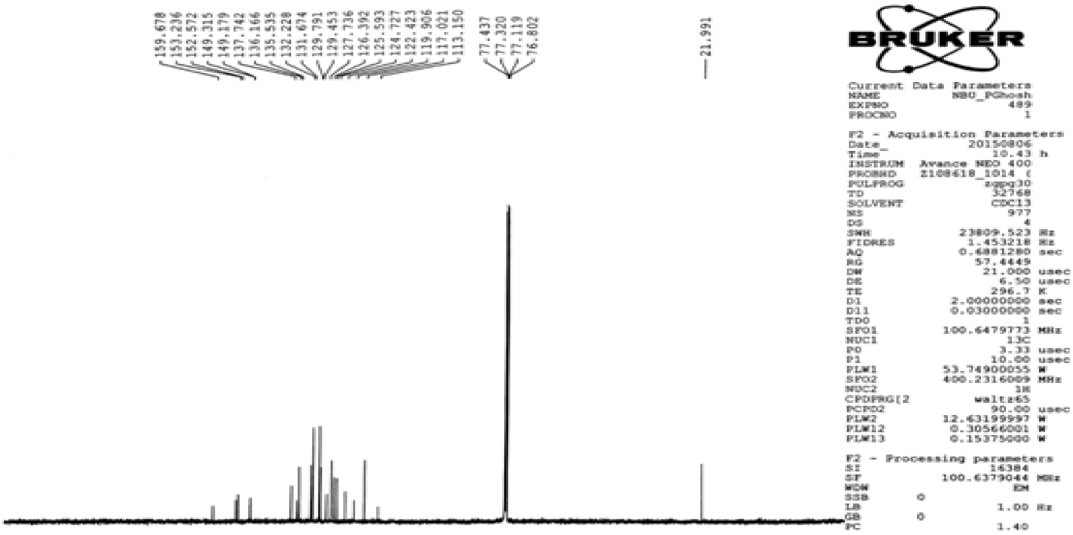

Fig 25. ^{13}C NMR spectrum of compound 4h

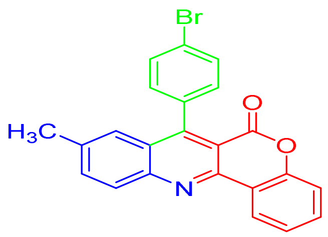

Fig 26. Structure of compound 4h

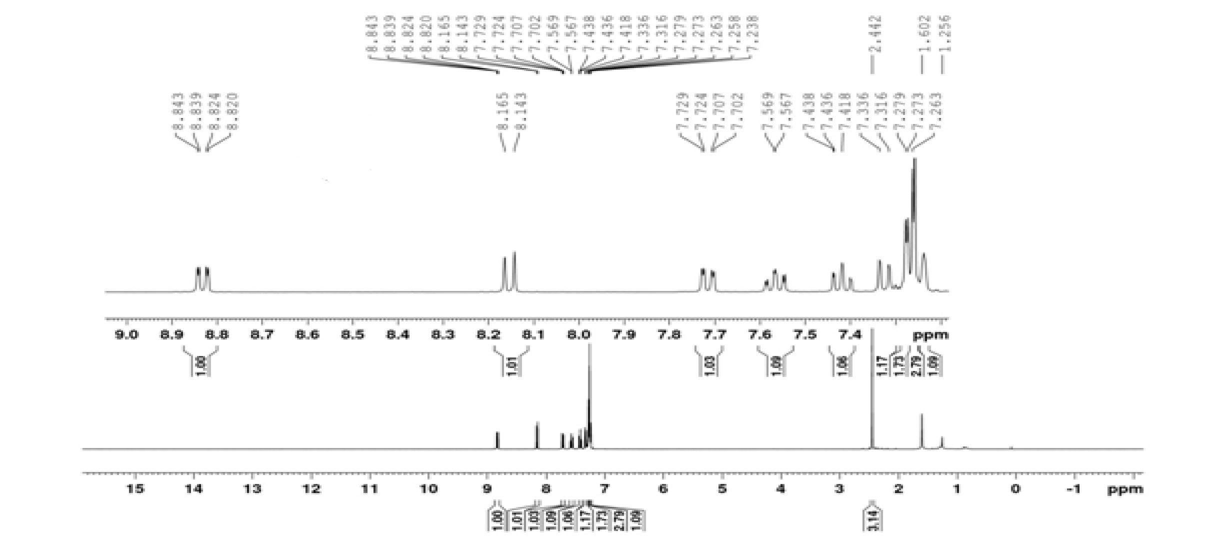

Fig 27. ^{1}H NMR spectrum of Compound 4i

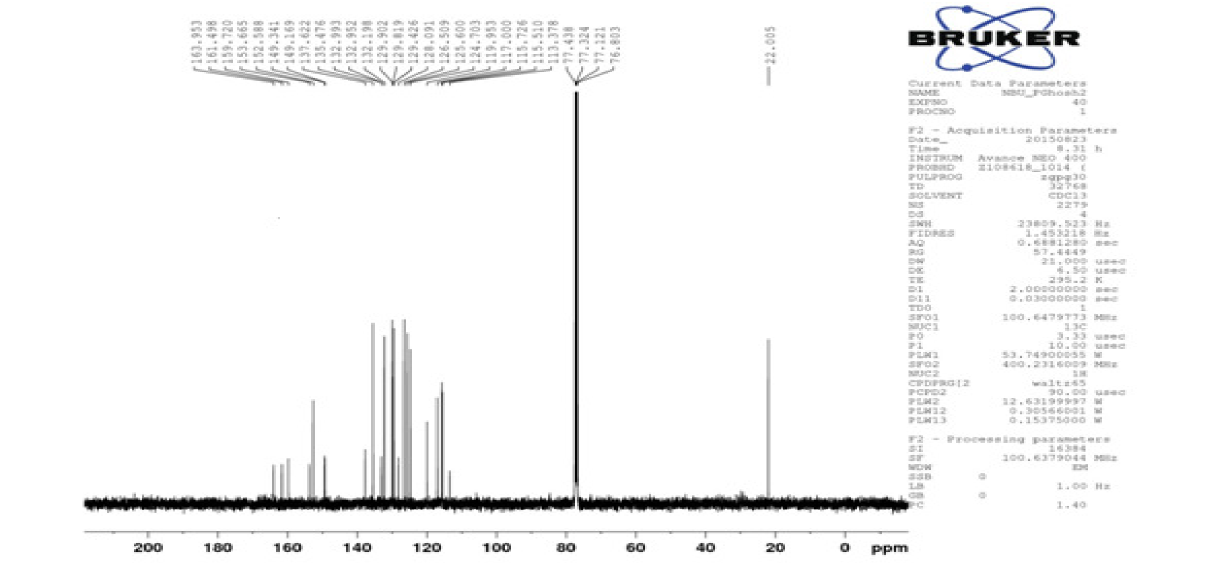

Fig 28. ^{13}C NMR spectrum of compound 4i

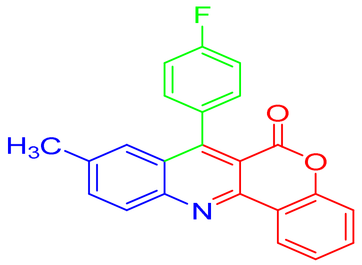

Fig 29. Structure of compound 4i

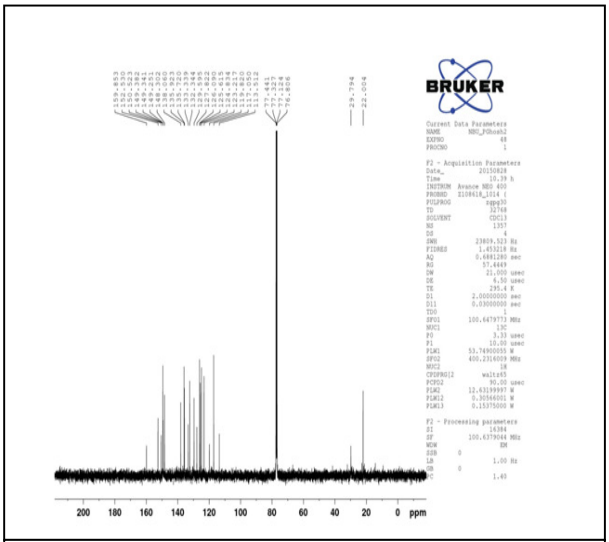

Fig 30. ^{13}C NMR spectrum of compound 4k

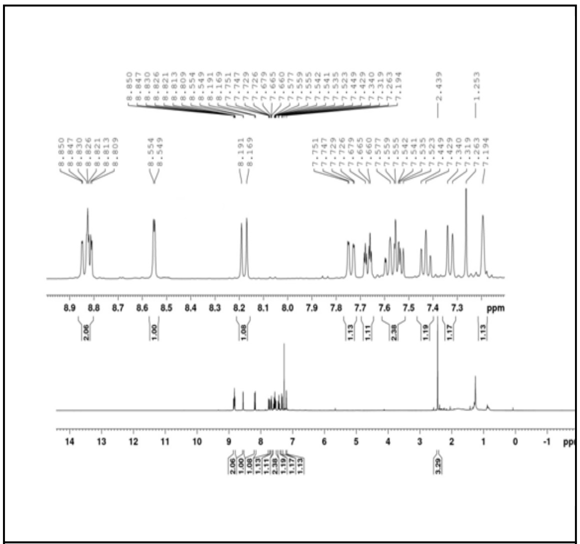

Fig 31. ^{1}H NMR spectrum of Compound 4k

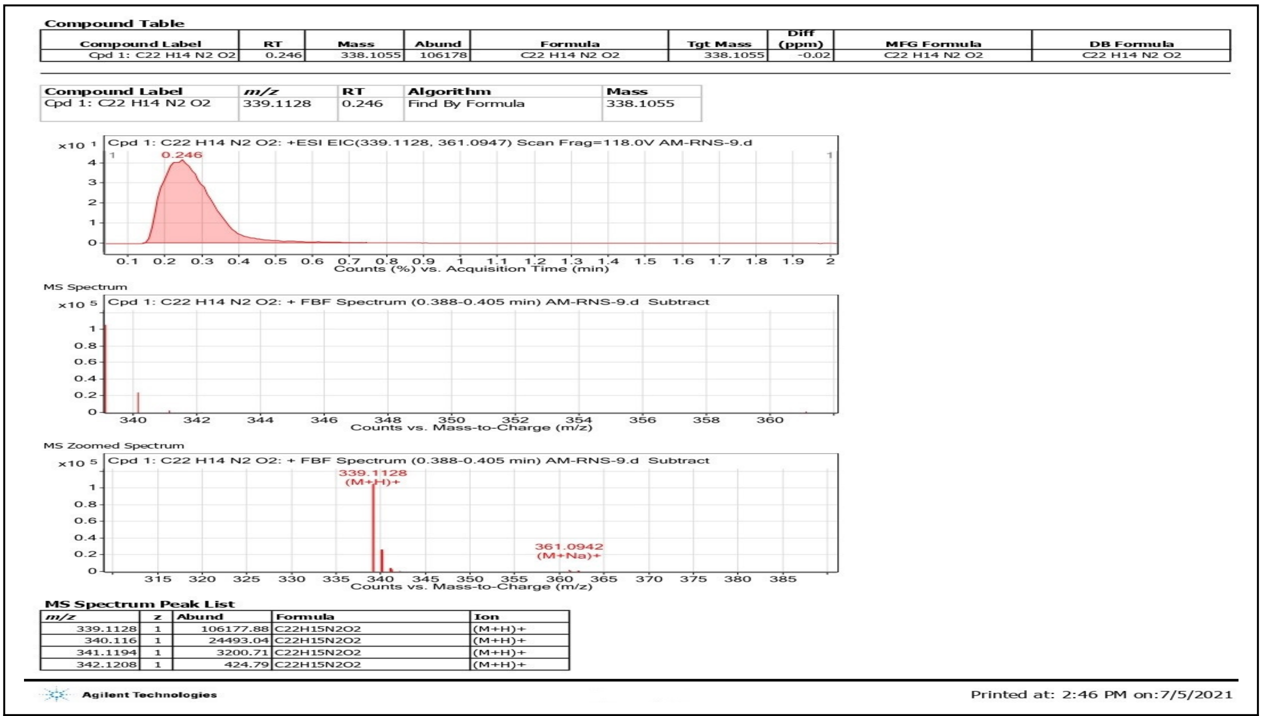

Compound Table

Compound Label	RT	Mass	Abund	Formula	Tgt Mass	Diff (ppm)	MFG Formula	DB Formula
Cpd 1: C22 H14 N2 O2	0.246	338.1055	106178	C22 H14 N2 O2	338.1055	-0.02	C22 H14 N2 O2	C22 H14 N2 O2

Compound Label	*m/z*	RT	Algorithm	Mass
Cpd 1: C22 H14 N2 O2	339.1128	0.246	Find By Formula	338.1055

MS Spectrum Peak List

m/z	z	Abund	Formula	Ion
339.1128	1	106177.88	C22H15N2O2	(M+H)+
340.116	1	24493.04	C22H15N2O2	(M+H)+
341.1194	1	3200.71	C22H15N2O2	(M+H)+
342.1208	1	424.79	C22H15N2O2	(M+H)+

Agilent Technologies Printed at: 2:46 PM on:7/5/2021

Fig 32. Mass spectrum of Compound 4k

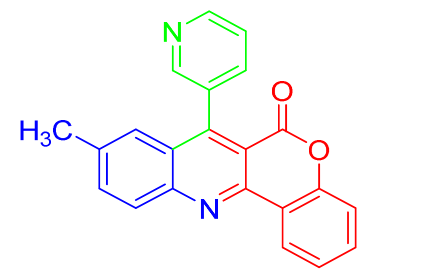

Fig 33. Structure of compound 4k

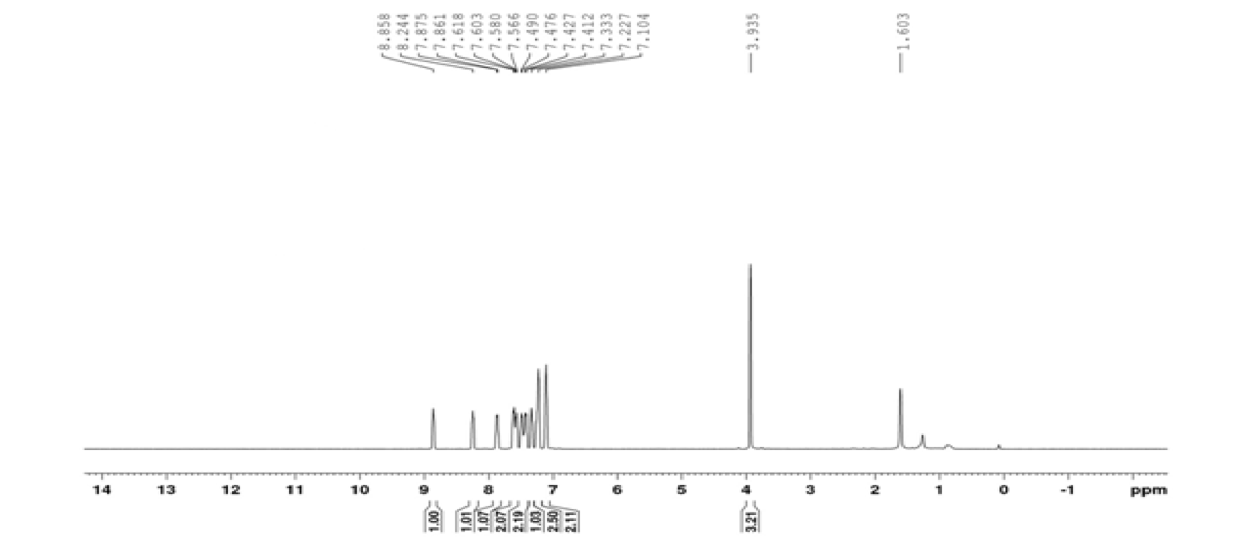

Fig 34. ^{1}H NMR spectrum of Compound 4l

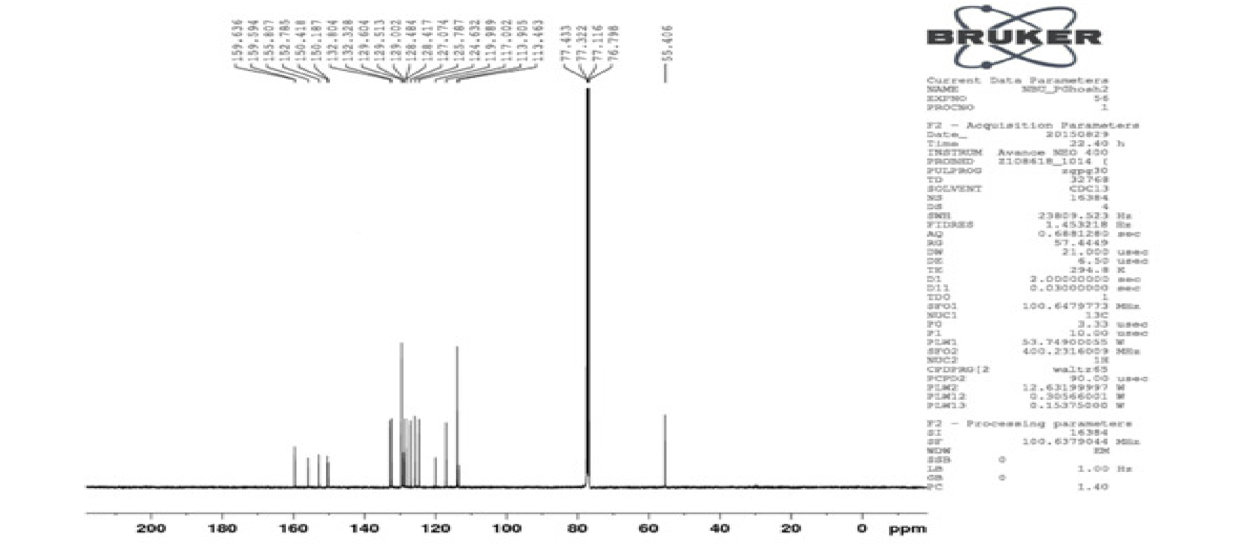

Fig 35. ^{13}C NMR spectrum of compound 4l

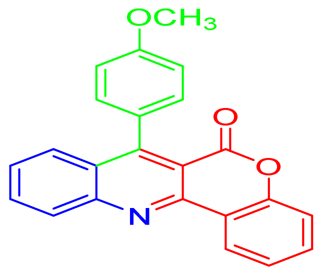

Fig 36. Structure of compound 4l

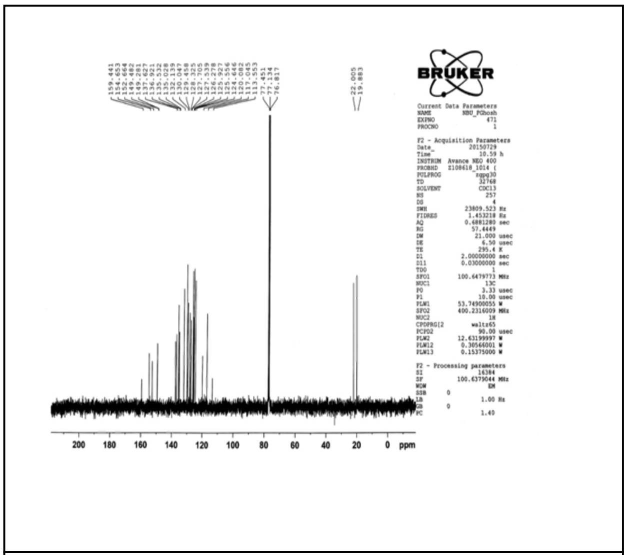

Fig 37. ^{13}C NMR spectrum of compound

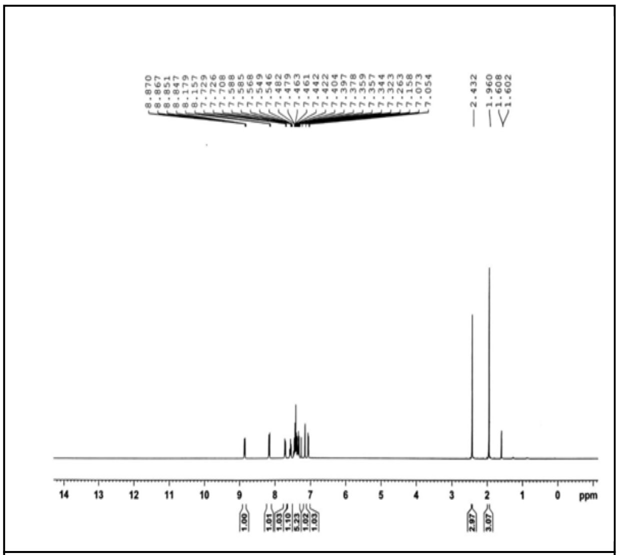

Fig 38. ^{1}H NMR spectrum of

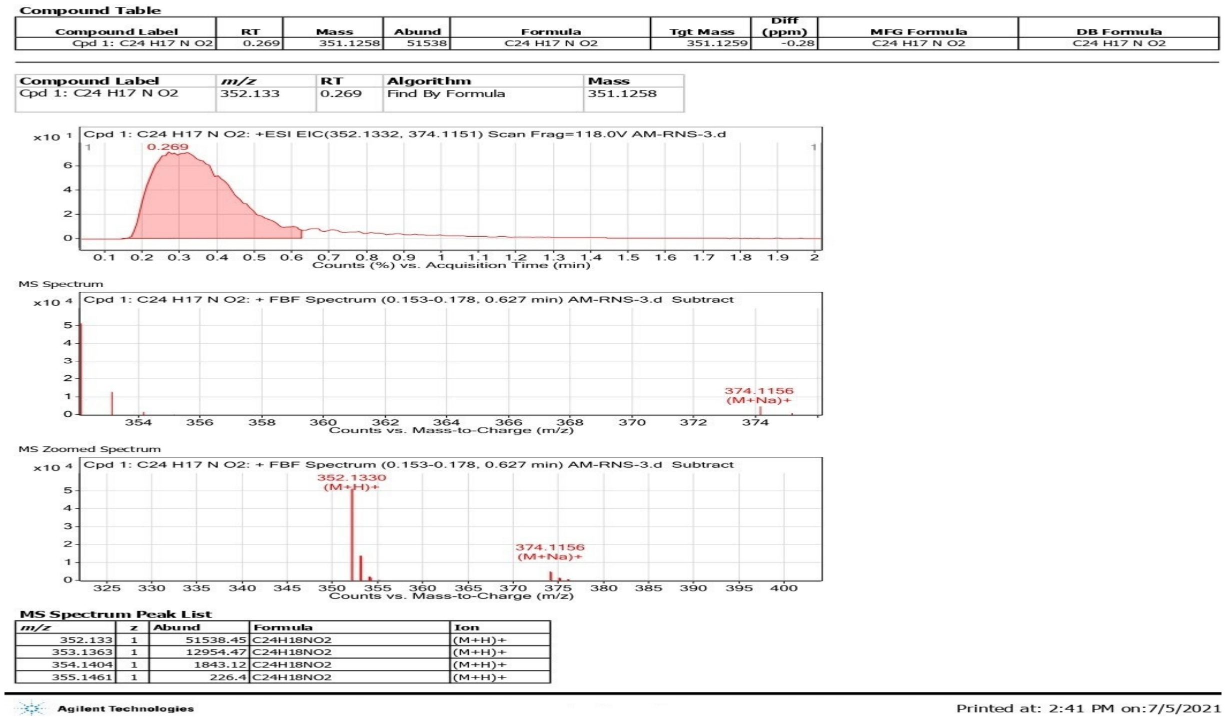

Compound Table

Compound Label	RT	Mass	Abund	Formula	Tgt Mass	Diff (ppm)	MFG Formula	DB Formula
Cpd 1: C24 H17 N O2	0.269	351.1258	51538	C24 H17 N O2	351.1259	-0.28	C24 H17 N O2	C24 H17 N O2

Compound Label	m/z	RT	Algorithm	Mass
Cpd 1: C24 H17 N O2	352.133	0.269	Find By Formula	351.1258

MS Spectrum Peak List

m/z	z	Abund	Formula	Ion
352.133	1	51538.45	C24H18NO2	(M+H)+
353.1363	1	12954.47	C24H18NO2	(M+H)+
354.1404	1	1843.12	C24H18NO2	(M+H)+
355.1461	1	226.4	C24H18NO2	(M+H)+

Agilent Technologies   Printed at: 2:41 PM on:7/5/2021

Fig 39. Mass spectrum of Compound 4m

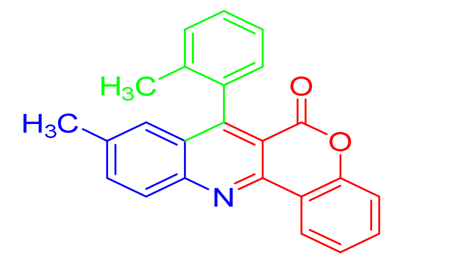

Fig 40. Structure of compound 4m

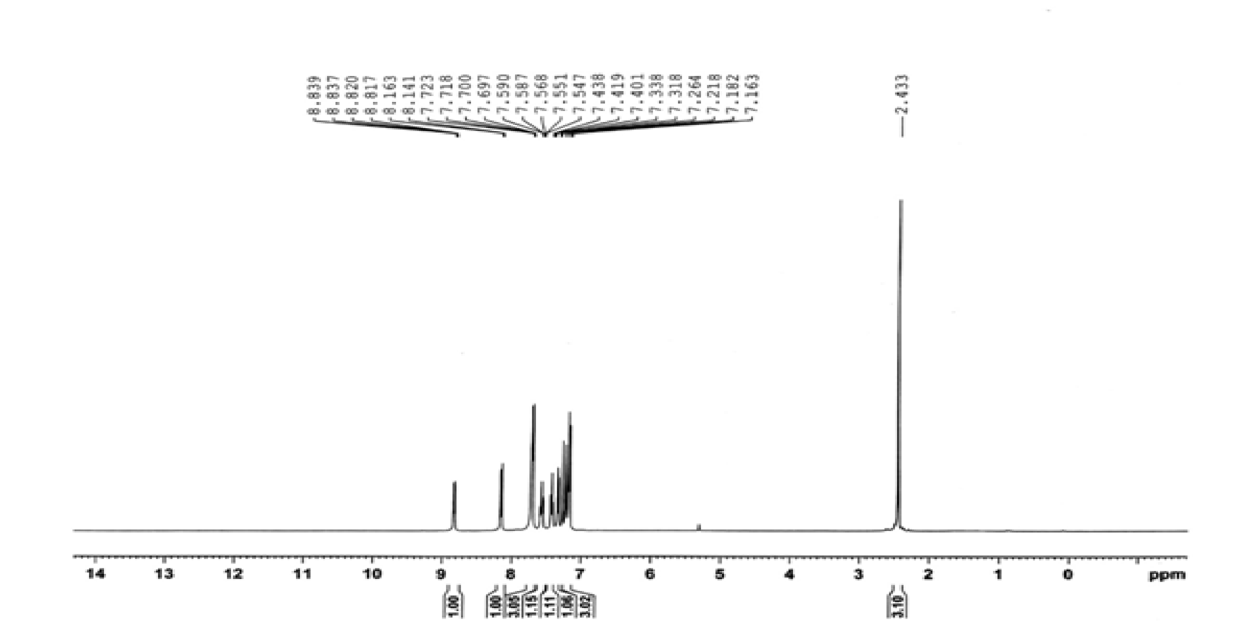

Fig 41. ^{1}H NMR spectrum of Compound 4n

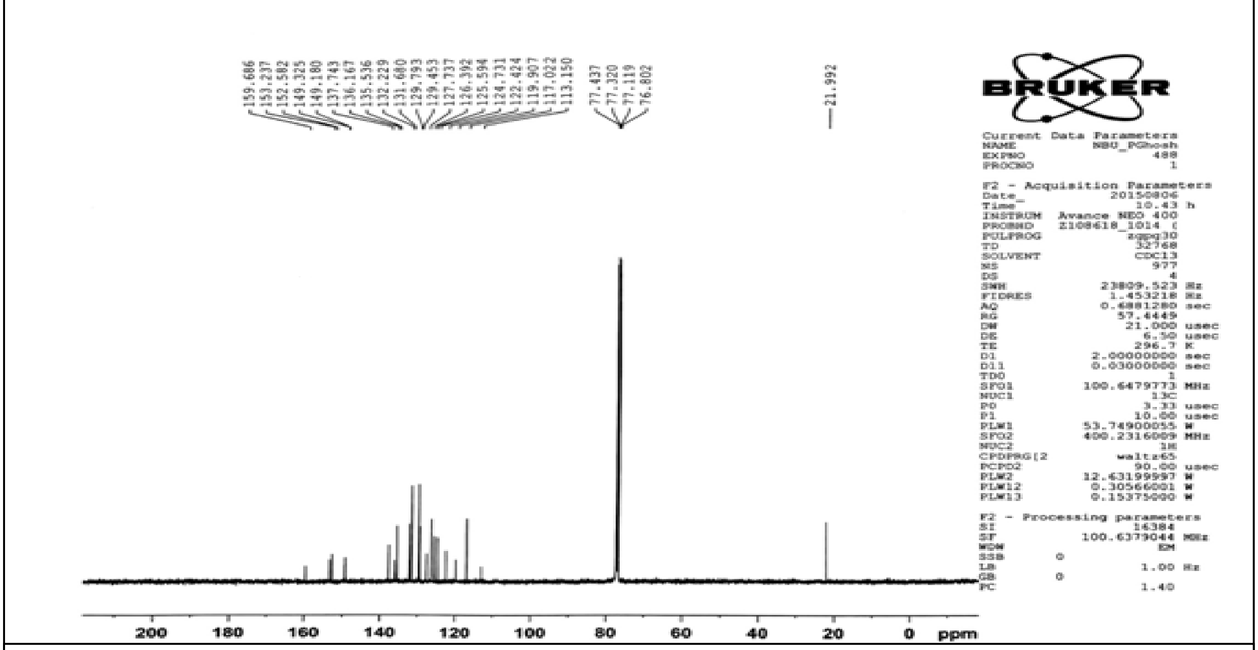

Fig 42. ^{13}C NMR spectrum of compound 4n

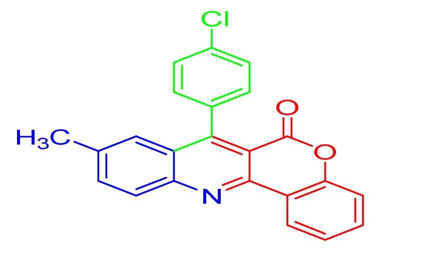

Fig 43. Structure of compound 4n

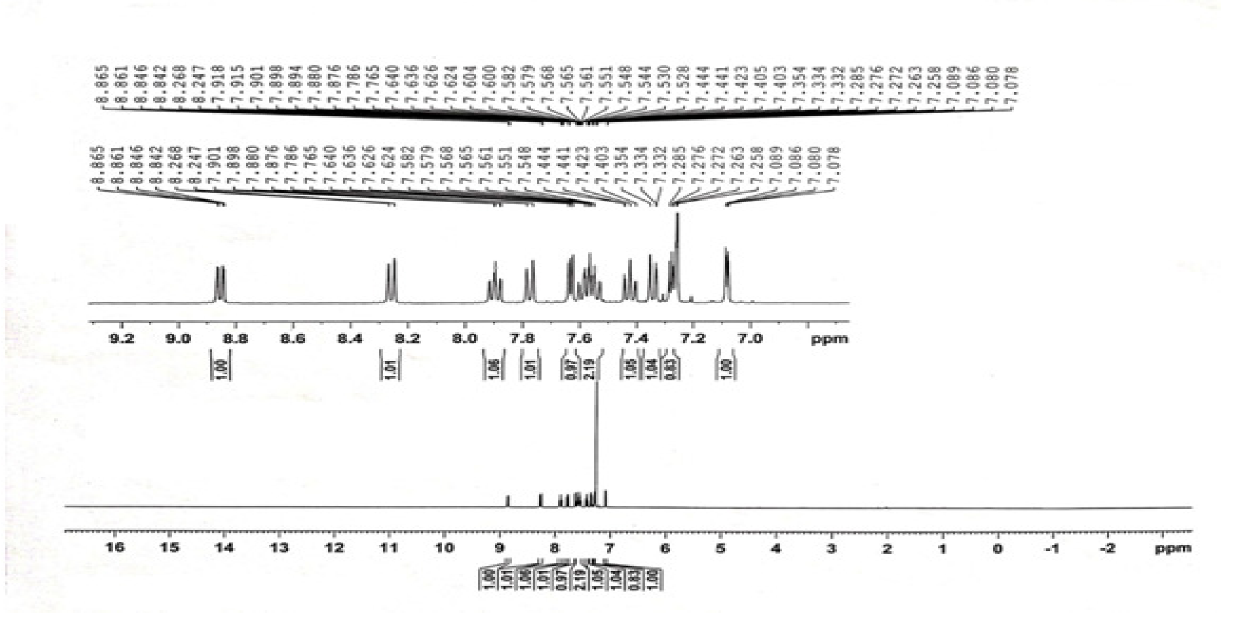

Fig 44. ^{1}H NMR spectrum of Compound 4o

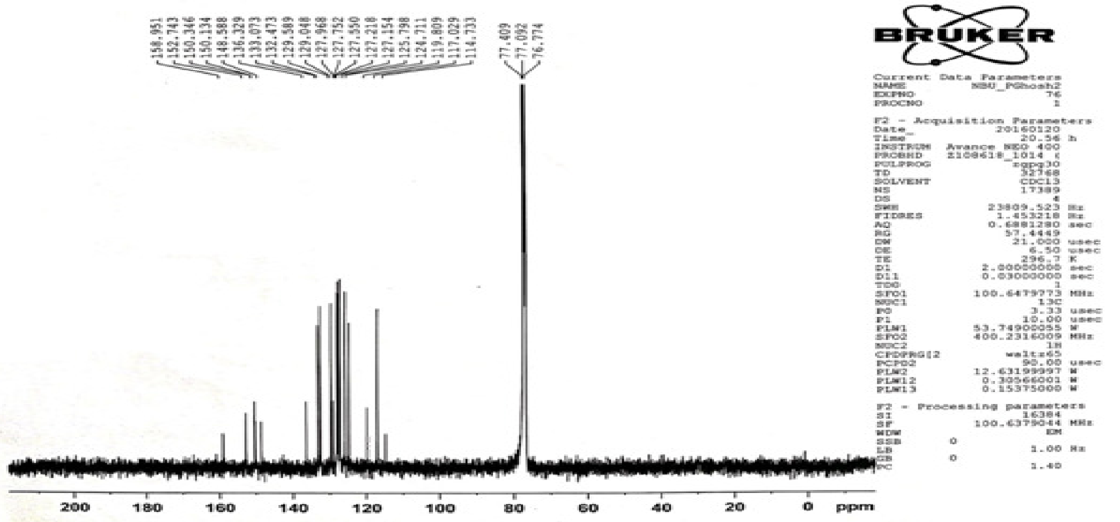

Fig 45. ^{13}C NMR spectrum of compound 4o

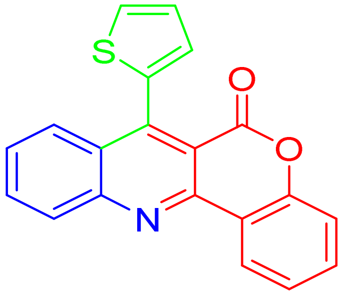

Fig 46. Structure of compound 4o

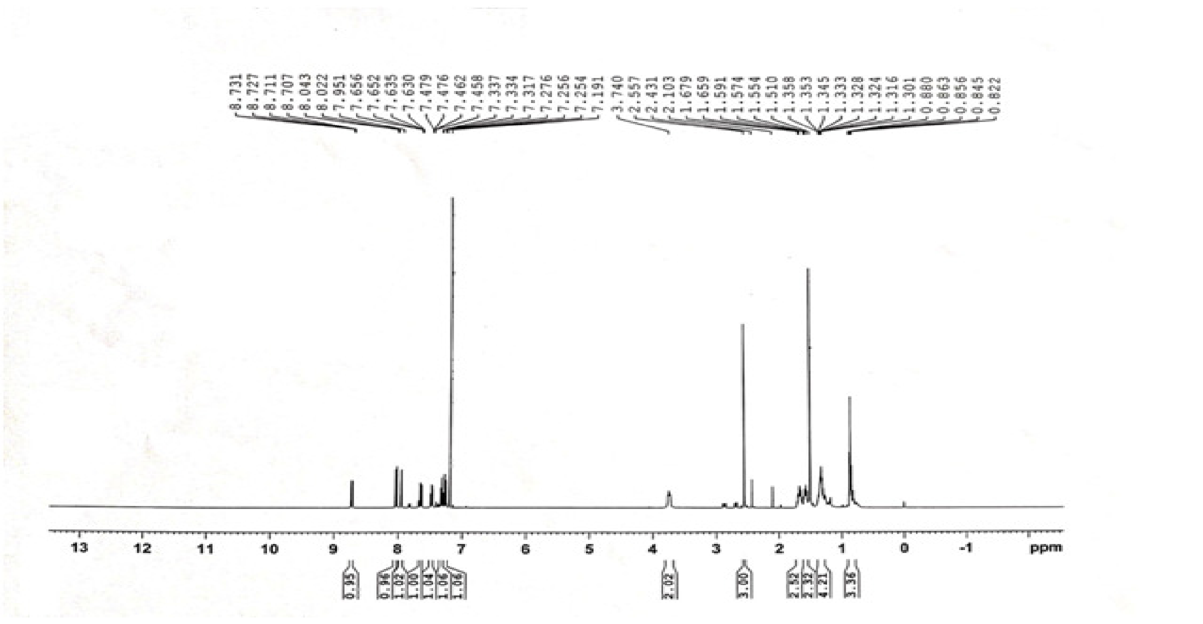

Fig 47. ^{1}H NMR spectrum of Compound 4p

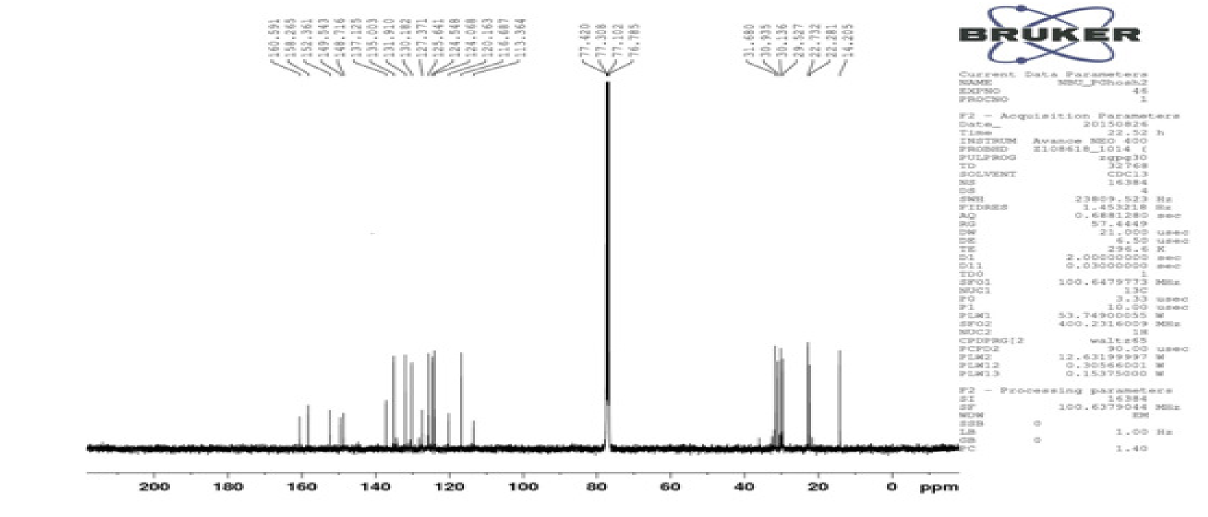

Fig 48. ^{13}C NMR spectrum of compound 4p

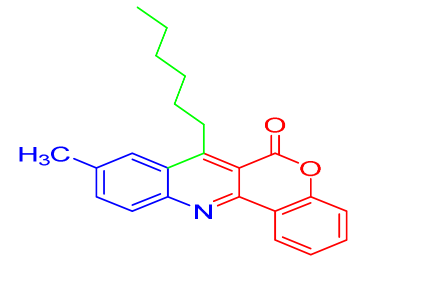

Fig 49. Structure of compound 4p
